# In vivo profiling of astrocyte secretome reveals brain-region specific regulatory networks in a mouse model of amyloid pathology

**DOI:** 10.1186/s13024-026-00956-y

**Published:** 2026-05-23

**Authors:** Qiu Jiang, Jian-Ni Hu, Hao-Min Dong, Jia-Yan Xin, An-Yu Shi, Gui-Hua Zeng, Jie Liu, Yan-Jiang Wang

**Affiliations:** 1 Department of Neurology and Centre for Clinical Neuroscience, Daping Hospital, Army Medical University (Third Military Medical University), Chongqing, 400042 China; 2 Chongqing Key Laboratory of Ageing and Brain Diseases, Chongqing, 400042 China; 3 Department of Cognitive Impairment Research, Chongqing Institute for Brain and Intelligence, Guangyang Bay Laboratory, Chongqing, 401336 China; 4 Shigatse Branch, Xinqiao Hospital, Army Medical University (Third Military Medical University), Tibet, 857012 China

**Keywords:** Alzheimer’s disease, Astrocyte, Secretome, Entorhinal cortex, Hippocampus, Early pathogenesis

## Abstract

Coordinated cell-to-cell communications is crucial for the proper functioning and maintenance of brain activities, and its disruption contributes to neurological disorders, including Alzheimer’s disease (AD). Altered astrocyte-neuron communications have been implicated in AD progression, yet the underlying regulatory networks remain poorly understood. Given that secretory proteins mediate both local and long-range intercellular signaling, we constructed a spatiotemporal profile of the astrocyte-derived secretome using in vivo TurboID proximity labeling in mice of amyloid pathology. Early alterations in the entorhinal cortex secretome were identified and enriched in metabolic pathways, whereas changes in the hippocampus were observed later, correlating with neuronal and synaptic maintenance. These findings suggest that early remodeling of the astrocyte secretome in the entorhinal cortex may be involved in AD pathogenesis, while later changes in the hippocampus contribute to neurodegeneration and cognitive decline. This work provides a systematic map of the dynamic, region-specific remodeling of the astrocyte secretome in AD, identifying novel spatiotemporal vulnerabilities and potential therapeutic targets.

**Supplementary Information:**

The online version contains supplementary material available at 10.1186/s13024-026-00956-y.

## Introduction

Alzheimer’s disease (AD), the most prevalent neurodegenerative disorder and a leading cause of dementia, imposes a substantial socioeconomic and caregiving burden worldwide [1]. Recent advances in omics technologies have shifted the focus from studies of isolated cell types toward network-level analyses aimed at elucidating the complex cellular interactions underlying AD pathology [2, 3]. Indeed, multi-omics studies, including transcriptomic [4] and proteomic [5] analyses coupled with network biology approaches, have revealed a widespread alternation in intercellular communication in the brains of AD patients, involved in Aβ clearance, neuroinflammation, and calcium homeostasis. Therefore, elucidating the aberrant intercellular communications during AD progression is pivotal to unraveling the core cellular network dynamics that orchestrate disease development.

Among the diverse cell types in the central nervous system, astrocytes have garnered significant attention for their multifaceted roles in neurodegenerative diseases [6]. In the context of AD, astrocytes exhibit complex, region- and stage-specific alterations in gene expression [7], and the dysregulation of glia-neuron interactions has increasingly been recognized as a key pathological feature. A primary mechanism for this communication is the secretion of proteins, including cytokines, chemokines, and growth factors, which orchestrate cellular responses. Therefore, profiling the secretome offers a powerful approach to systematically map astrocyte-mediated intercellular signaling in AD, providing a mechanistic framework for understanding their contribution to pathology.

However, the majority of studies on the astrocyte secretome have relied on in vitro models [8–10]. These models, while valuable, lack the intricate cellular milieu and signaling networks of the in vivo brain environment, potentially yielding profiles that do not fully represent physiological or pathological states. In vivo secretome analysis, on the other hand, presents a formidable challenge: isolating proteins secreted by a specific cell type from the complex extracellular environment. The recent advent of enzyme-mediated proximity labeling technologies provides a powerful solution to this problem. Specifically, TurboID, an engineered biotin ligase with high catalytic efficiency, enables the rapid (within ~ 10 min) and spatially-restricted (~ 10 nm radius) biotinylation of proximal proteins [11]. This technique has been successfully applied to map the secretomes of various cell types in vivo, including in the liver [12] and muscle [13], leading to significant biomarker discoveries and mechanistic insights.

In this study, we utilized TurboID-based proximity labeling coupled with mass spectrometry (MS) to characterize the astrocyte-derived secretome in vivo across two key brain regions at distinct pathological stages in a mouse model of amyloid pathology. Our objective is to generate a dynamic, spatiotemporal map of astrocytic communication networks throughout AD progression. By doing so, this work provides novel insights into the region-specific cellular events underlying AD pathogenesis and offers a valuable resource for the identification of new biomarkers and therapeutic targets.

## Results

### TurboID-based proximity labeling for the in vivo astrocyte secretome

**Fig. 1:**
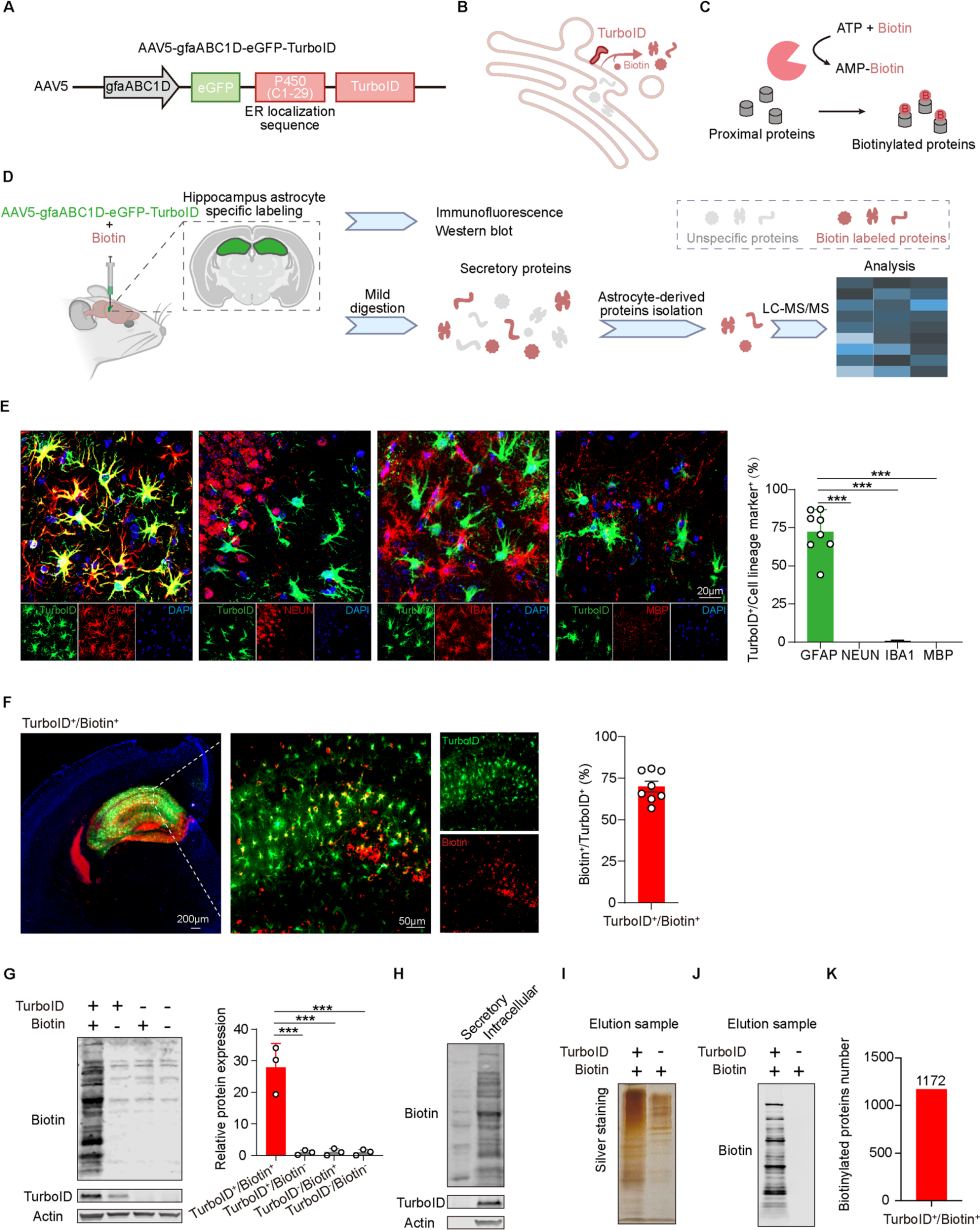
TurboID-based proximity labeling for the in vivo astrocyte secretome. **A**. Design of viral vectors. The virus specifically targets astrocytes, enabling cell-specific expression of TurboID. **B.** TurboID is localized to the ER, enabling preferential biotinylation of proteins undergoing classical secretion. **C.** Mechanism of action of TurboID. TurboID catalyzes ATP and biotin into the reactive intermediate AMP-Biotin, which covalently tags proximal proteins. **D.** Technical flowchart of the pilot study. Following viral injection into the hippocampus of WT mice and biotin (5 mg/mL) administration, the expression specificity and biotinylation efficiency of TurboID were evaluated. Concurrently, the hippocampal tissues of five mice were pooled into one sample, from which biotinylated secretory proteins were extracted and enriched for downstream mass spectrometry analysis. **E.** Representative images show that TurboID specifically targets astrocytes, with minimal co-localization observed in other cell types (*n* = 8 mice, 2–3 images per mouse were averaged to yield a single value per mouse). One-way ANOVA tests were used, ***p < 0.001. **F.** Representative images demonstrating efficient TurboID-mediated biotinylation and labeling efficiency (*n* = 8 mice, 2–3 images per mouse were averaged to yield a single value per mouse). **G.** TurboID specificity for proteins biotinylation in the hippocampus. Biotin signal in TurboID^+^/Biotin^+^ group was significantly higher than that in the control groups (*n* = 3 biological replicates, each replicate was pooled from the hippocampal tissues of 5 mice). TurboID^+^/Biotin^+^ group: AAV5-gfaABC1D-eGFP-TurboID expression followed by biotin administration; TurboID^+^/Biotin^−^ group: AAV5-gfaABC1D-eGFP-TurboID expression followed by PBS administration; TurboID^−^/Biotin^+^ group: AAV5-gfaABC1D-eGFP expression followed by biotin administration; TurboID^−^/Biotin^−^ group: AAV5-gfaABC1D-eGFP expression followed by PBS administration. One-way ANOVA tests were used, ***p < 0.001. **H.** Extraction of secretory proteins in the hippocampus. Compared with intracellular proteins, secretory proteins showed absence of TurboID and Actin. **I.** Silver staining of the magnetic bead eluted sample from TurboID^+^/Biotin^+^ and TurboID^−^/Biotin^+^ group. **J.** Specific biotin signals were detected in the magnetic bead elution samples from TurboID^+^/Biotin^+^ group, but not in TurboID^−^/Biotin^+^ group. **K.** Preliminary mass spectrometry analysis of number of astrocyte-derived secretory proteins in TurboID^+^/Biotin^+^ group. ER, endoplasmic reticulum; LC/MS, Liquid Chromatograph/Mass Spectrometer; WT, wild type

To achieve astrocyte-specific proteins labeling, we engineered an AAV5 vector carrying endoplasmic reticulum (ER)-localized TurboID (ER-TurboID) under the control of the gfaABC1D promoter (AAV5-gfaABC1D-eGFP-TurboID) (Fig. 1A). The gfaABC1D promoter is an engineered, truncated derivative of the GFAP promoter (~ 680 bp) that has been extensively optimized to achieve a balance between strong expression and astrocyte specificity [14]. ER-TurboID preferentially labels most classical secretory proteins transiting through the ER-Golgi pathways (Fig. 1B), has been applied in in vivo studies of tissue- or cell type-specific secretomes [13, 15]. TurboID catalyzes ATP and biotin into the reactive intermediate AMP-Biotin, which covalently tags proximal proteins (Fig. 1C) [15].

We first validated our system in wild-type (WT) mice by stereotactically injecting AAV5-gfaABC1D-eGFP-TurboID into the hippocampus. Following one month of viral expression, biotin was administered in situ for 5 consecutive days. During technological exploration, the biotinylation efficiency in the 5-day administration group reached 77%, significantly higher than 40% in the 3-day group (Fig. S1A). Secretory proteins were then extracted from fresh hippocampal tissue. Then, biotinylated secretory proteins were enriched using streptavidin magnetic beads for MS analysis (Fig. 1D). Immunofluorescence staining confirmed that TurboID expression was highly specific to astrocytes, co-localizing with ~ 72% of GFAP-positive cells and showing no overlap with markers for neurons (NEUN), microglia (IBA1), or oligodendrocytes (MBP) (Fig. 1E).

To assess the efficiency and specificity of biotinylation, we established four groups: one experimental group (TurboID^+^/Biotin^+^), in which AAV5-gfaABC1D-eGFP-TurboID was expressed followed by biotin administration, and three negative control groups, including TurboID^+^/Biotin^−^ (AAV5-gfaABC1D-eGFP-TurboID expression followed by PBS administration), TurboID^−^/Biotin^+^ (AAV5-gfaABC1D-eGFP expression followed by biotin administration), and TurboID^−^/Biotin^−^ (AAV5-gfaABC1D-eGFP expression followed by PBS administration). As expected, a robust biotin signal, with ~ 70% labeling efficiency, was detected exclusively in the TurboID^+^/Biotin^+^ group (Fig. 1F and S1B). Western blot also confirmed the presence of biotinylated protein bands only in this group (Fig. 1G).

Secretory proteins were extracted using a mild digestion procedure that enzymatically degrades extracellular matrix collagen without disrupting cellular integrity. This method gently releases secretory proteins from the intercellular space and is widely used for enriching extracellular vesicles from brain tissue [16]. This approach successfully isolated secretory proteins, as biotinylated proteins were detected in the extract, whereas intracellular proteins such as TurboID and Actin were absent (Fig. 1H) [17]. Subsequent enrichment with streptavidin beads found that TurboID^−^/Biotin^+^ control group contained low-abundance non-specific background signals (Fig. 1I), while the TurboID^+^/Biotin^+^ experimental group exhibited specific high-abundance biotinylated protein bands (Fig. 1J). To minimize the influence of nonspecific proteins binding during the enrichment process, the TurboID^−^/Biotin^+^ group was used as a technical control. Proteins showing more than a twofold higher abundance in the TurboID^+^/Biotin^+^ group compared with the TurboID^−^/Biotin^+^ group were defined as astrocyte-derived secretory proteins [18]. This analysis identified 1,172 astrocyte-derived secretory proteins, including known astrocyte-enriched proteins like GFAP and GLUL (Fig. 1K).

Collectively, these results demonstrate that our AAV-TurboID platform enables efficient and specific labeling and enrichment of the astrocyte-derived secretome in vivo, providing a robust tool for investigating astrocytic communication.

### Spatiotemporal profiling of the astrocyte secretome in an AD mouse model

**Fig. 2:**
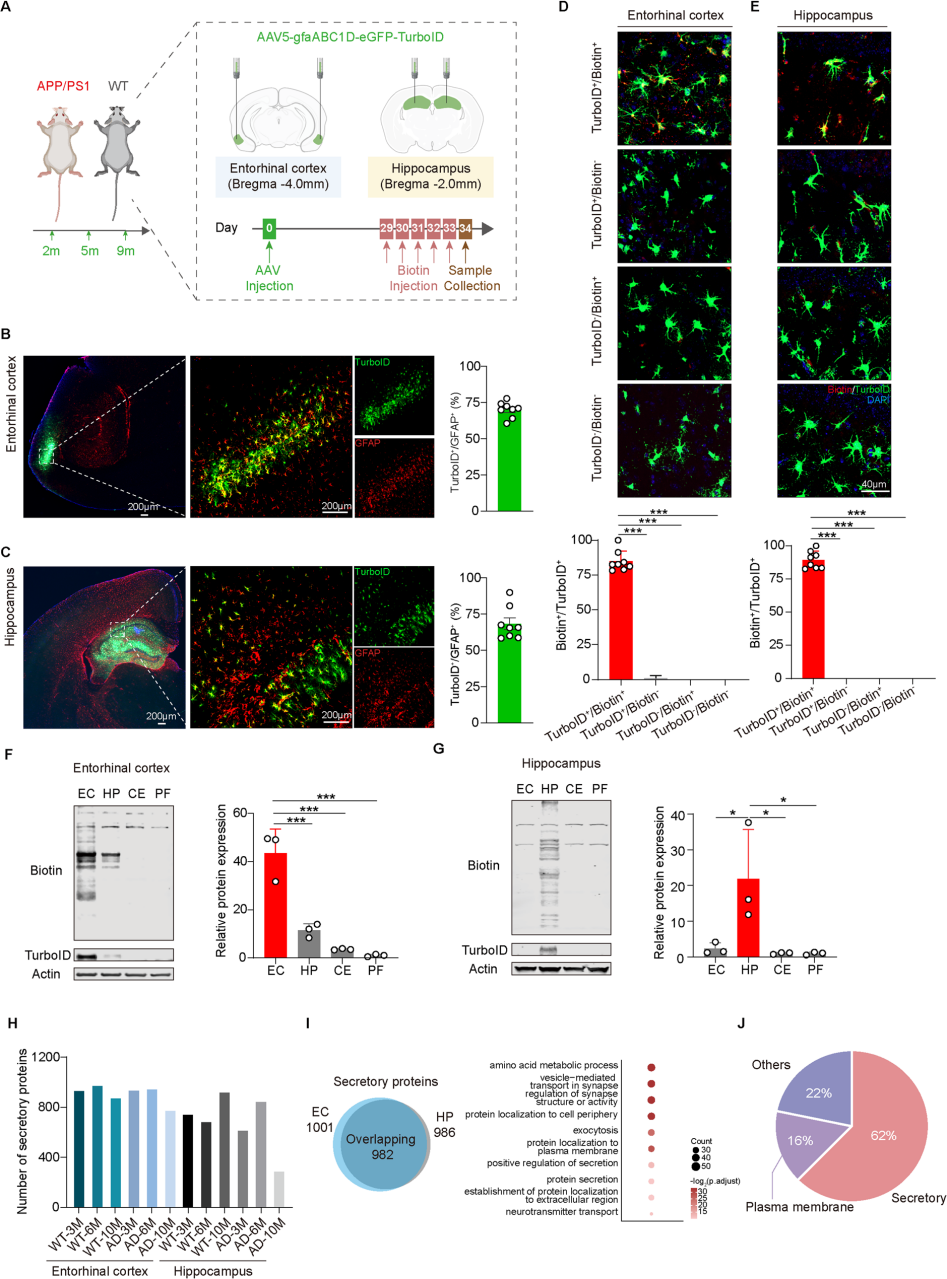
Profiling of astrocyte-derived secretory proteins in the entorhinal cortex and hippocampus. **A.** Schematic illustration of the mouse model. Briefly, AAV5-gfaABC1D-eGFP-TurboID was stereotactically injected into the entorhinal cortex and hippocampus of *APP/PS1* and age-matched WT mice at 2, 5, and 9 months of age. One month after viral injection, biotin (5 mg/mL) was administered daily for five consecutive days (TurboID⁺/Biotin⁺). Tissue samples were collected 24 h after the final dose for subsequent analysis. **B**, **C**. Representative images showing the astrocyte-specific expression of TurboID and the efficiency of astrocyte targeting in the entorhinal cortex and hippocampus (*n* = 8 mice, 2–3 images per mouse were averaged to yield a single value per mouse). **D**, **E**. Representative images showing the efficiency of TurboID-mediated protein biotinylation in the entorhinal cortex and hippocampus. Biotin signal in TurboID^+^/Biotin^+^ group was significantly higher than that in the control groups (*n* = 8 mice, 2–3 images per mouse were averaged to yield a single value per mouse). TurboID^+^/Biotin^+^ group: AAV5-gfaABC1D-eGFP-TurboID expression followed by biotin administration; TurboID^+^/Biotin^−^ group: AAV5-gfaABC1D-eGFP-TurboID expression followed by PBS administration; TurboID^−^/Biotin^+^ group: AAV5-gfaABC1D-eGFP expression followed by biotin administration; TurboID^−^/Biotin^−^ group: AAV5-gfaABC1D-eGFP expression followed by PBS administration. One-way ANOVA tests were used, ***p < 0.001. **F**, **G**. Brain region-specific protein biotinylation. Biotinylation signals were markedly enriched in the virus-injected regions and were significantly lower in non-injected areas (*n* = 3 biological replicates, each replicate was pooled from the tissues of 5 mice). One-way ANOVA tests were used, *p < 0.05, ***p < 0.001. **H**. The number of astrocyte-derived secretory proteins across different experimental groups. Astrocyte-derived secretory proteins were screened by comparing the experimental group (AAV5-gfaABC1D-eGFP-TurboID expression followed by biotin administration) to the technical control group (AAV5-gfaABC1D-eGFP expression followed by biotin administration), using a threshold of *p* < 0.05 and log₂FC > 1. An unpaired two-tailed Student’s t-test was used for statistical analysis. *n* = 3 biological replicates in mass spectrometry; each replicate was pooled from the entorhinal cortex or hippocampal tissues of 5 mice. **I**. Venn diagram of astrocyte-derived secretory proteins of entorhinal cortex and hippocampus respectively, and biological process of overlapping proteins. **J**. Subcellular localization of all astrocyte-derived secretory proteins. WT, wild type; EC, entorhinal cortex; HP, hippocampus; CE, cerebellum; PF, prefrontal cortex; FC, fold change

We next applied this platform to delineate the dynamic remodeling of the astrocyte secretome throughout AD progression. Utilizing a mouse model of amyloid pathology (*APP/PS1* mice), we targeted three distinct time points: 3 months (early Aβ deposition), 6 months (intermediate amyloid deposition), and 10 months (late amyloid deposition) [19]. Our investigation focused on the entorhinal cortex (the primary site of early pathology) and the hippocampus (a region critical for memory and susceptible to progressive atrophy). To characterize age- and AD-associated alterations in the astrocyte secretome, we stereotactically injected AAV5-gfaABC1D-eGFP-TurboID into the bilateral entorhinal cortex or hippocampus of *APP/PS1* mice and age-matched WT mice at 2, 5, and 9 months, allowing for one month of viral expression prior to biotin administration and analysis (TurboID^+^/Biotin^+^) (Fig. 2A). TurboID^−^/Biotin^+^ served as the technical control group, in which AAV5-gfaABC1D-eGFP and biotin were administered to the hippocampus of 2-month-old WT mice to exclude nonspecific background proteins. Detailed experimental conditions for all groups are provided in the Methods.

TurboID was stably and specifically expressed in astrocytes of both the entorhinal cortex and hippocampus, with an efficiency of ~ 70% (Fig. 2B, C). Robust biotinylation (~ 80% efficiency) was observed exclusively in the TurboID^+^/Biotin^+^ groups in both regions (Fig. 2D, E, and S1C). To assess the regional distribution of biotinylated proteins, tissue samples were collected from the entorhinal cortex, hippocampus, cerebellum, and prefrontal cortex. The biotin signal was predominantly confined to the targeted region, confirming the spatial specificity of our approach (Fig. 2F, G). Intracellular protein contamination was also confirmed to be negligible (Fig. S1D).

For proteomic profiling, hippocampal or entorhinal cortical tissues from five mice were pooled to constitute one biological replicate. Three independent biological replicates were prepared for each condition, namely, the TurboID⁺/Biotin⁺ experimental group and the TurboID⁻/Biotin⁺ control group and analyzed by LC-MS/MS, yielding an effective sample size of *n* = 3 independent pools per condition. From the entorhinal cortex and hippocampus of both *APP/PS1* and WT mice at three time points, we identified a total of 1007 astrocyte-derived secretory proteins (*p* < 0.05 and log_2_FC > 1 vs. TurboID^−^/Biotin^+^ control group), with over 600 proteins detected in most groups (Fig. 2H). The entorhinal cortex and hippocampus contained 1001 and 986 proteins, respectively, with a 98% overlap (982 proteins), indicating high similarity in the composition of the astrocyte secretome between these regions (Fig. 2I). Astrocyte-derived secretory proteins constituted 10–15% of the total secretory proteins (Fig. S2), lower than astrocyte abundance in the brain (20–40%) [20, 21]. This discrepancy likely stems from astrocyte labeling efficiency (~ 72%) and the inability of TurboID to capture unconventionally secreted proteins (~ 22–26% of the secretome) [22].

Gene Ontology (GO) analysis of the shared proteins revealed enrichment in functions such as synaptic transport, extracellular protein localization, exocytosis, and neurotransmitter balance (Fig. 2I), consistent with known astrocyte functions [23]. Subcellular localization analysis showed that these identified proteins were highly enriched for classical secretory (62%) and plasma membrane-associated (16%) proteins [24], accounting for 78% of the total—a significant enrichment over the ~ 34% baseline proportion of secretory proteins in the proteome [25] (Fig. 2J). This demonstrates the high efficiency of our method in capturing the secretome. The remaining ~ 22% of proteins likely include those released via non-classical secretion pathways, such as direct translocation or lysosome-mediated secretion [26], which are not annotated as secretory in current databases. Unconventional secretion is increasingly recognized, particularly under activated or pathological conditions [27].

### Region-specific alterations of the astrocyte secretome during normal aging

**Fig. 3:**
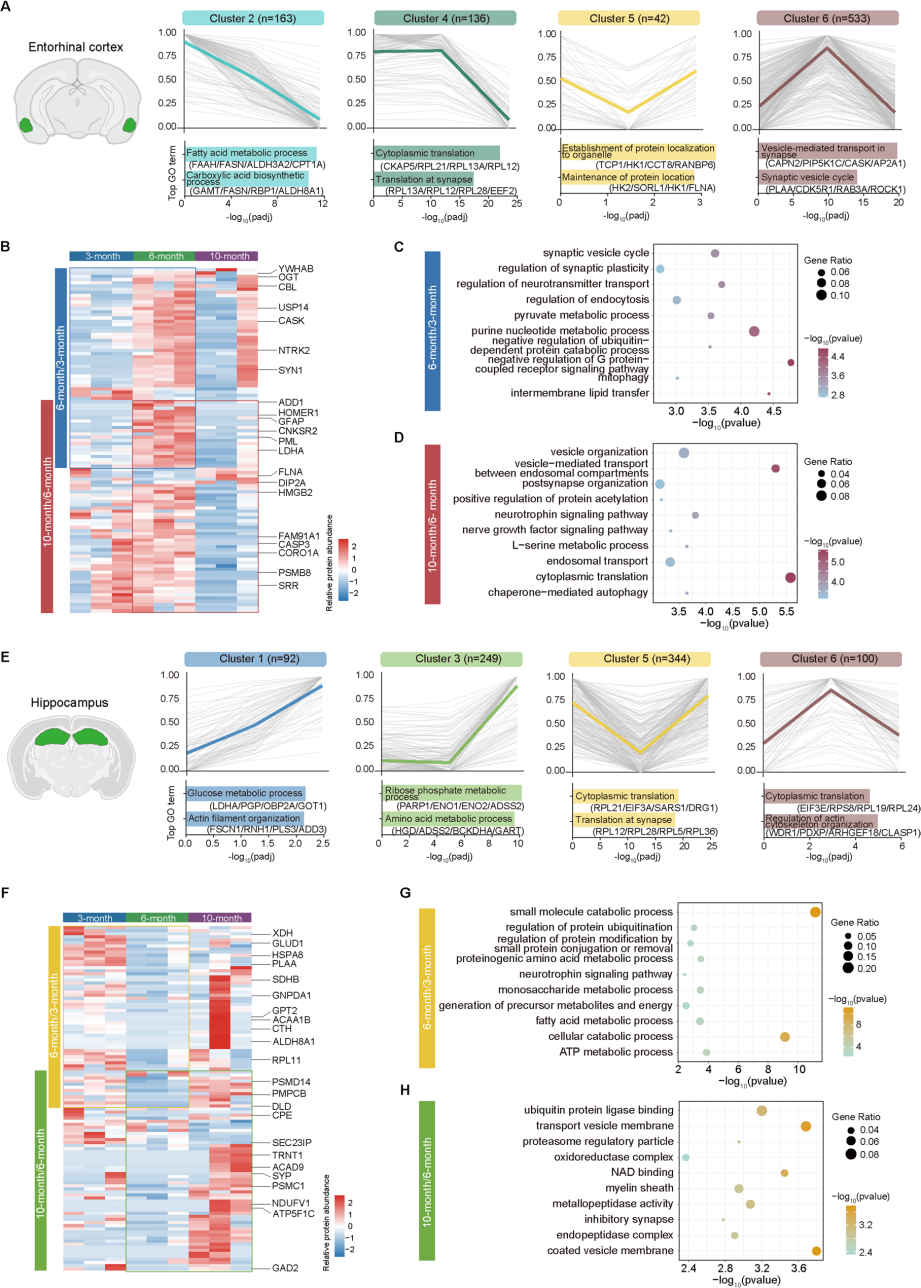
Alterations of astrocyte-derived secretory proteins during aging. **A**. Dynamic alterations of astrocyte-derived secretory proteins in the entorhinal cortex of WT mice. Total secretory proteins were classified into nine distinct clusters based on their changing trends, four of which and their corresponding GO terms were presented here. **B**. Heatmap of DEPs (*p* < 0.05) at different ages in the entorhinal cortex of WT mice. Proteins associated with aging and AD are specifically highlighted on the right. An unpaired two-tailed Student’s t-test was used for statistical analysis. *n* = 3 biological replicates; each replicate was pooled from the entorhinal cortex tissues of 5 mice. **C**, **D**. The GO terms of biological processes corresponding to the DEPs from 3 to 6 months (**C**) and 6 to 10 months (**D**) respectively in the entorhinal cortex. **E**. Dynamic alterations of astrocyte-derived secretory proteins in the hippocampus of WT mice. Total secretory proteins were classified into nine distinct clusters based on their changing trends, four of which and their corresponding GO terms were presented here. **F**. Heatmap of DEPs (*p* < 0.05) at different ages in the hippocampus of WT mice. Proteins associated with aging and AD are specifically highlighted on the right. An unpaired two-tailed Student’s t-test was used for statistical analysis. *n* = 3 biological replicates; each replicate was pooled from the hippocampal tissues of 5 mice. **G**, **H**. The GO terms corresponding to the DEPs from 3 to 6 months (**G**) and 6 to 10 months (**H**) respectively in the hippocampus. WT, wild type; GO, gene ontology; DEPs, differentially expressed proteins

To establish a pathological baseline, we first analyzed the astrocyte secretome dynamics in WT mice during normal aging (3 to 10 months). Strikingly, we observed complementary, region-specific temporal trajectories between the entorhinal cortex and hippocampus (Fig. 3A, E). The remaining clusters are presented in the supplementary materials (Fig. S3).

In the entorhinal cortex, the period from 3 to 6 months was characterized by a dramatic upregulation of secretome activity, with 94% of differentially expressed proteins (DEPs) being elevated (Fig. 3B). Functional enrichment analysis revealed that these changes were strongly associated with synaptic plasticity and neural development (Fig. 3C). Specifically, key regulators of synaptic formation and maturation, such as NTRK2 and SYN1 [28, 29], were significantly upregulated, suggesting an age-related promotion of neural network development by astrocyte-derived secretory proteins. Conversely, the hippocampus displayed an inverse pattern during the same period, with 95% of DEPs being decreased (Fig. 3F). These downregulated proteins were primarily related to metabolic pathways, including glucose, ATP, and fatty acid metabolism (Fig. 3G). The decrease in GLUD1, an essential protein for inhibitory synapse formation in pyramidal neurons [30], indicated an early compromise in astrocytic metabolic and energetic support to neurons in the hippocampus.

The trend reversed in the later stage. From 6 to 10 months, the entorhinal cortex showed an age-related decline in secretome activity, evidenced by the downregulation of 92% of DEPs (Fig. 3D). These proteins were mainly related to protein transport and synthesis. Among these, the neuroprotective HOMER1 was downregulated. HOMER1 has been shown to stabilize reactive astrocytes and inhibit neuroinflammation [31], suggesting that its downregulation led to a loss of neuro-supportive roles in the entorhinal cortex. Simultaneously, the hippocampus exhibited a significant increase in secretome activity, with 83% of DEPs being upregulated (Fig. 3F). These increased proteins were linked to vesicle membrane components and ubiquitin protein ligase binding, affecting protein transport and degradation pathways (Fig. 3H). Notably, TRNT1 was upregulated, a change closely associated with the production of inflammatory factors, reactive oxygen species (ROS), and dysregulation of protein degradation pathways [32].

These complementary, region-specific patterns of astrocyte-derived secretory proteins observed in the entorhinal cortex and hippocampus during aging may stem from the existence of distinct astrocytic subtypes. Single-cell sequencing studies have, in fact, delineated region-specific astrocyte subtypes, revealing a clear molecular and functional segregation between cortical and hippocampal populations [33]. Thus, these astrocytic subtypes exhibited heterogeneous dynamics during aging.

### Spatiotemporal dysregulation of the astrocyte secretome during AD progression

**Fig. 4:**
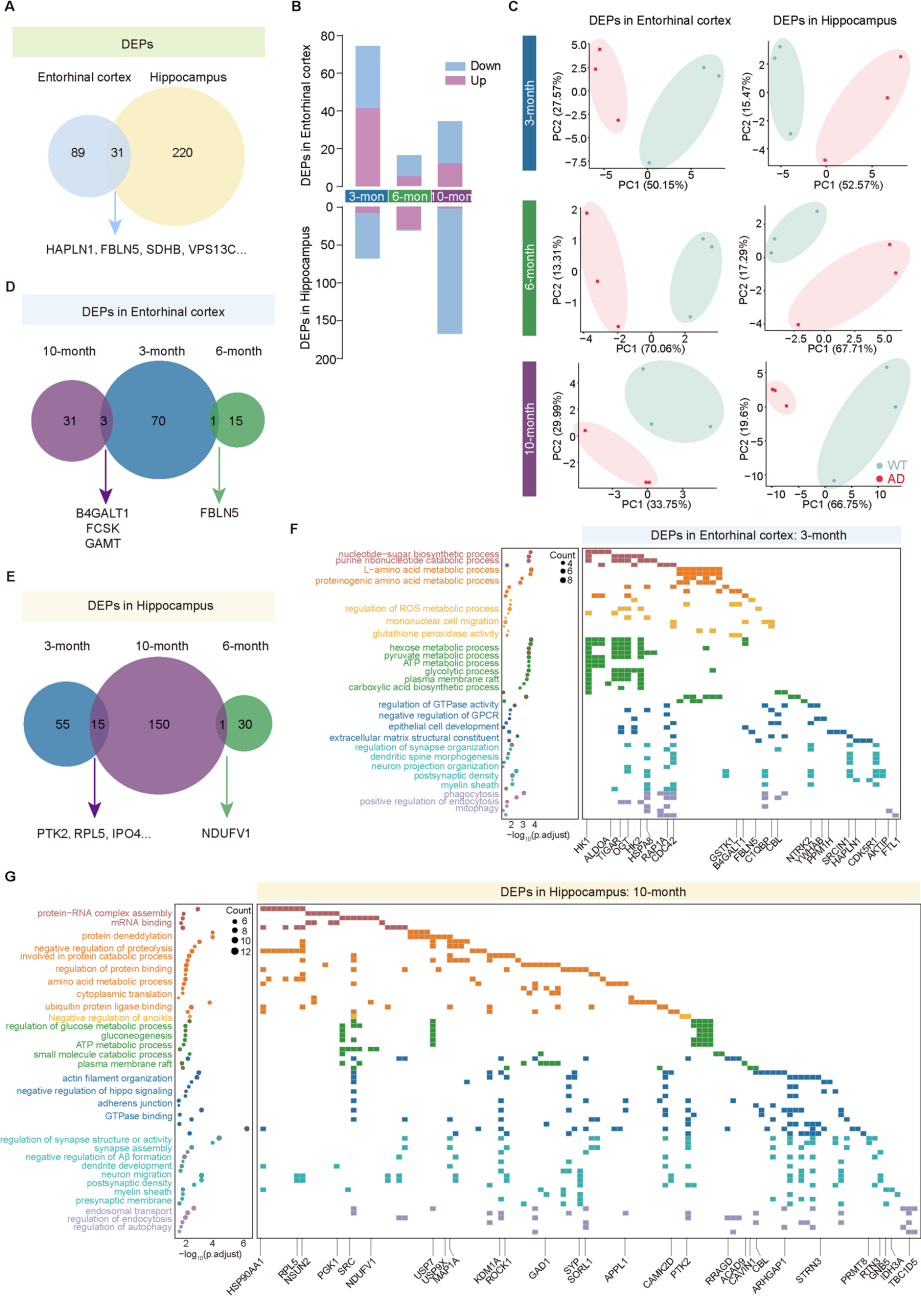
Screening age-stage-specific DEPs in AD progression. **A**. Comparison of DEPs in the entorhinal cortex and hippocampus respectively. DEPs were identified by comparing age-matched *APP/PS1* mice and WT mice, performing an unpaired two-tailed Student’s t-test in combination with FC value (*p* < 0.05 and |log_2_FC|>1). *n* = 3 biological replicates in mass spectrometry, each replicate was pooled from the entorhinal cortex or hippocampal tissues of 5 mice. **B**. Numbers of DEPs identified in each age-stage along with the counts of upregulated and downregulated DEPs respectively. **C**. The principal component analysis of identified DEPs in Fig. 4B. The APP/PS1 mice were clearly distinguished from WT mice in both the entorhinal cortex and hippocampal tissues across all three time points. **D**, **E**. Venn diagram illustrating the number of overlapping DEPs across different age-stages in the entorhinal cortex (**D**) and hippocampus (**E**). **F**, **G**. GO terms of DEPs in the 3-month entorhinal cortex (**F**) and 10-month hippocampus (**G**). Pathways associated with AD were identified and classified into seven functional modules. These pathways and their corresponding DEPs were presented. The p values of the GO terms listed on the left. The listed biological processes were highlighted with red circles. DEPs linked to AD were marked below. Dot size indicates the number of proteins enriched in the corresponding biological process. DEPs, differentially expressed proteins; FC, fold change; WT, wild type; GO, gene ontology

We next compared the astrocyte secretomes of *APP/PS1* mice with age-matched WT controls, 340 DEPs were identified (*p* < 0.05 and |log_2_FC|>1). Of these, 120 were in the entorhinal cortex, and 251 in the hippocampus, with 31 overlapping (Fig. 4A). In the context of Aβ pathology, the number of DEPs peaked at 3 months in the entorhinal cortex and at 10 months in the hippocampus, further pinpointing the entorhinal cortex as the site of early astrocytic dysregulation (Fig. 4B). Principal component analysis (PCA) of these identified DEPs confirmed a clear separation between WT and *APP/PS1* groups in all stages (Fig. 4C). However, across different stages, the overlap in DEPs between *APP/PS1* and WT mice was minimal (Fig. 4D, E). In the entorhinal cortex, FBLN5 was upregulated at 3 and 6 months and overexpressed in reactive astrocytes [34, 35], suggesting an early astrocytic shift toward a pro-inflammatory and neurotoxic phenotype. At 3 and 10 months, B4GALT1 was downregulated, modulating *APP* processing [36]. In the hippocampus, among the 15 overlapping DEPs at 3 and 10 months, downregulation of PTK2 impaired microglial chemotactic migration toward Aβ deposits, exacerbating Aβ burden [37]. At 6 and 10 months, only NDUFV1 was common. Its reduced secretion in late-stage AD impairs proteasomal degradation, promoting pathological proteins accumulation [38].

Functional enrichment analysis revealed distinct pathological themes: early changes in the entorhinal cortex were predominantly metabolic, involving proteins linked to oxidative stress and Aβ neurotoxicity, e.g., TIGAR, HSPA8 and HK2 [39–41] (Fig. 4F). Late changes in the hippocampus were related to impaired neuronal development and synaptic plasticity, with downregulation of key proteins like RTN3 and SORL1, which are critical for processing of APP and clearance of protein aggregates [42, 43] (Fig. 4G). Heatmap of DEPs (Fig. S4) and pathway enrichment results for DEPs from other stages in the entorhinal cortex and hippocampus (Fig. S5) are provided in the supplementary materials.

To further elucidate the transcriptional dynamics underlying these astrocyte-related pathways, we analyzed the mRNA expression of DEPs implicated in key pathological pathways, including metabolism, autophagy, Aβ processing, and regulation of synaptic and neuronal activity. We verified using 3-, 6-, and 10-month *APP/PS1* and WT mice (*n* = 8 for each group), and extracted entorhinal cortex (Fig. S6A) and hippocampus (Fig. S6B) tissues for reverse transcription quantitative PCR (RT-qPCR). In the entorhinal cortex, the transcript level of HSPA8 was elevated at 3 months, consistent with the astrocyte secretome. Previous studies have suggested that HSPA8 may promote the aggregation of Aβ monomers into oligomers [44]. In contrast, our results indicate that HSPA8 exerts direct neurotoxic effects, as in vitro treatment of primary mouse neurons with recombinant HSPA8 induced concentration-dependent neurotoxicity, characterized by marked simplification of dendritic arborization and increased neuronal cell death (Fig. S6C, D). In the hippocampus, downregulation of RTN3 and SORL1 transcript was also observed at 10 months. This coordinated decrease promotes amyloidogenesis through distinct mechanisms: loss of RTN3 enhanced BACE1 stability and enzymatic activity, fostering Aβ production [45], while reduced SORL1 disrupted lysosomal clearance, further exacerbating plaque deposition [42]. These transcriptomic findings reinforce the central role of astrocyte secretome in AD pathogenesis in context of Aβ pathology, demonstrating that astrocytic contributions are not only integral to the molecular cascades related to both early metabolic imbalance and late synaptic decline, but also affect pathological proteins dynamics and neuronal activity.

### Astrocyte secretome links aging to exacerbated AD pathogenesis

We compared DEPs in aging and AD to elucidate the molecular links between aging and AD (based on the Aβ pathology). In the entorhinal cortex, 21 overlapping proteins were identified and 70 were detected in the hippocampus, substantially more than in the entorhinal cortex (Fig. S7A). This may reflect that entorhinal cortex is an early-affected region in AD with weaker links to aging, whereas hippocampus is more severely impacted in late AD and closely tied to aging. In the entorhinal cortex, CP increased during aging but rose early and more highly in AD. Astrocytic CP knockdown reduced iron-induced oxidative stress and neuronal death in aged mice [46], suggesting astrocyte-derived CP may lead to neurotoxicity. In the hippocampus, RPS6, regulating proteins translation, decreased with aging and drop further in AD (Fig. S7B). Aβ-exposed astrocyte released extracellular vesicles enriched with RPS6 and actively delivered them to neuronal axons, modulating proteins synthesis and preserving synaptic structure and function. Conversely, diminished RPS6 secretion impaired axonal local translation and attenuated synapse-protective effects [47]. These findings suggest that aging-related changes in the astrocyte secretome are exacerbated in AD, leading to earlier and more severe pathology. Additionally, AD-specific DEPs (LRRC59, VPS13C) and aging-specific DEPs (GLTP, XDH) were identified in the entorhinal cortex and hippocampus (Fig. S7C, D), indicating distinct responses: the former linked to amyloid-associated changes and the latter to age-related functional decline.

### Early astrocyte secretome changes in the entorhinal cortex are involved in AD initiation

**Fig. 5:**
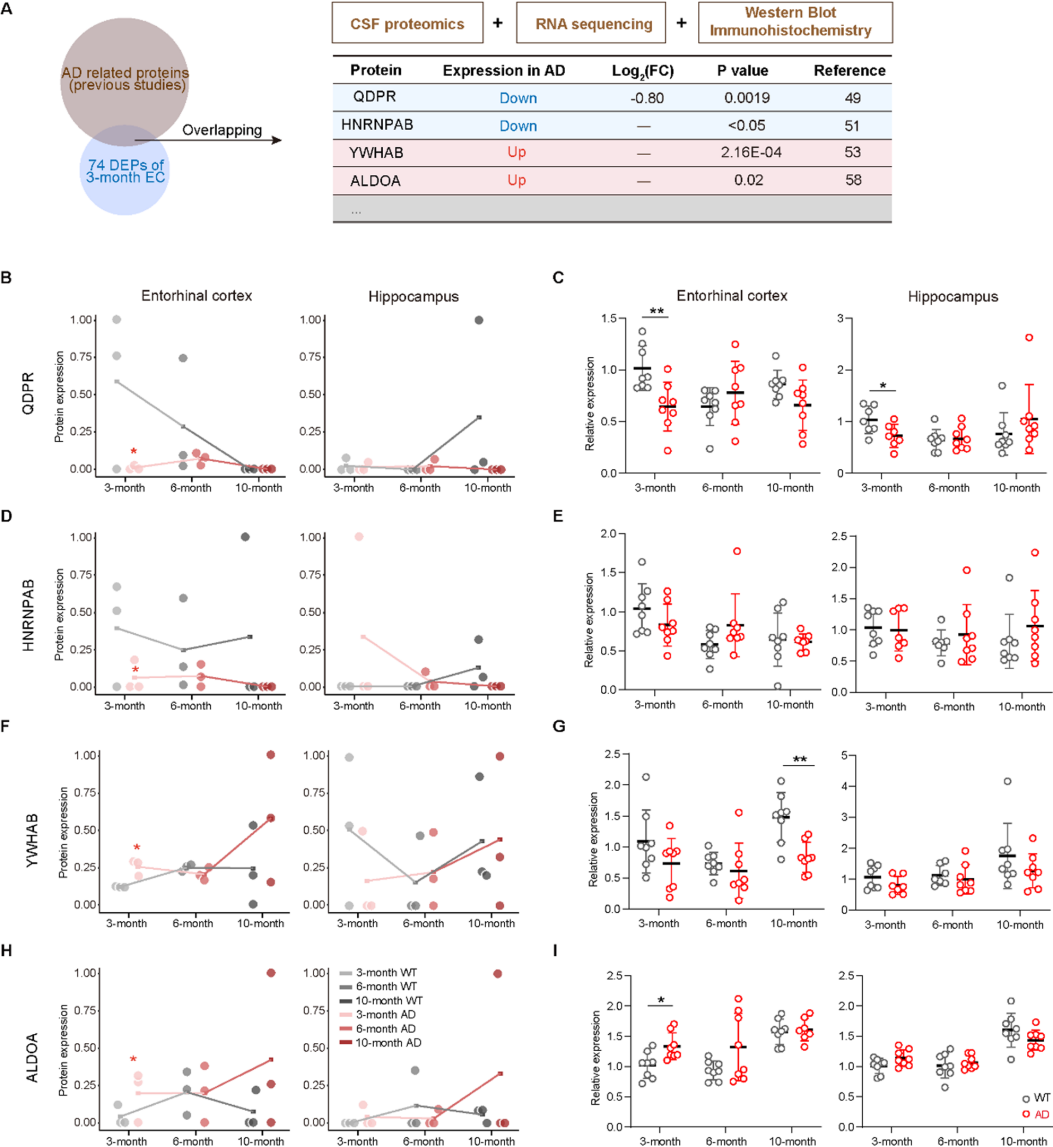
DEPs in the early entorhinal cortex involved in AD pathogenesis. **A**. Comparison of astrocyte-derived DEPs identified in this study with differentially expressed proteins/genes reported in previous AD omics studies. For the overlapping proteins, we focused on four DEPs in the 3-month entorhinal cortex, all of which exhibited expression trends consistent with previous studies. Blue shading indicates downregulation, while red shading indicates upregulation. **B**, **D**, **F**, **H**. In the entorhinal cortex and hippocampus, expression of astrocyte-derived DEPs, including QDPR (**B**), HNRNPAB (**D**), YWHAB (**F**) and ALDOA (**H**), in the *APP/PS1* and WT mice across different ages (*n* = 3 biological replicates in mass spectrometry, each replicate was pooled from the entorhinal cortex or hippocampal tissues of 5 mice). An unpaired two-tailed Student’s t-test and FC values were used, *p < 0.05 and |log_2_FC| > 1. **C**, **E**, **G**, **I**. In the entorhinal cortex and hippocampus, the mRNA expression levels corresponding to astrocyte-derived DEPs, including *Qdpr* (**C**), *Hnrnpab* (**E**), *Ywhab* (**G**) and *Aldoa* (**I**), in the *APP/PS1* and WT mice across different ages (*n* = 8 mice in RT-qPCR). An unpaired two-tailed Student’s t-test was used, *p < 0.05, **p < 0.01. AD, Alzheimer’s disease; DEPs, differentially expressed proteins; EC, entorhinal cortex; CSF, cerebrospinal fluid; FC, fold change; WT, wild type

To elucidate the mechanisms underlying the early astrocytic contribution to AD, we focused on DEPs from the 3-month entorhinal cortex that have established relevance in human AD studies. We selected four prominent candidates for detailed validation: two downregulated (QDPR, HNRNPAB) and two upregulated (YWHAB, ALDOA) (Fig. 5A).

#### Downregulated secretory proteins are implicated in early metabolic and synaptic stress

We first examined the downregulated proteins. QDPR, an enzyme critical for neurotransmitter synthesis and antioxidant defense [48], is known to be impaired in AD brains [49]. Our analysis revealed that astrocyte-derived QDPR was significantly decreased in the entorhinal cortex of 3-month-old AD mice, a change that was not observed in the hippocampus at this stage. A decline emerged in the hippocampus at 10 months (Fig. 5B). The reduction at the protein level in the 3-month entorhinal cortex was further supported by a corresponding decrease in *Qdpr* mRNA (Fig. 5C). To assess its functional relevance, primary neurons were treated with increasing concentrations of recombinant QDPR. This treatment resulted in a significant increase in dopamine production, a trend toward reduced ROS levels and improved cell viability, indicating a potential protective effect of QDPR (Fig. S8A, B).

Another protein is HNRNPAB, an RNA-binding protein [50] whose loss in the human entorhinal cortex was linked to dendritic damage and cognitive deficits [51]. Although predominantly a neuronal protein, we found that astrocyte-secreted HNRNPAB was significantly and persistently downregulated in the entorhinal cortex of AD mice from 3 months onward. In contrast, its levels in the hippocampus showed a transient increase before declining at 10 months (Fig. 5D). This was mirrored by a specific downregulation of *Hnrnpab* mRNA in the 3-month entorhinal cortex (Fig. 5E).

#### Upregulated secretory proteins point to pathogenic signaling and biomarker potential

Next, we analyzed the upregulated DEPs. YWHAB, a member of the 14-3-3 protein family, is one of the earliest biomarkers to rise in the cerebrospinal fluid (CSF) of AD patients, about 22-years before clinical onset [52, 53]. Our results show a concordant increase in astrocyte-secreted YWHAB in the 3-month entorhinal cortex (Fig. 5F). Interestingly, while this secretory protein was upregulated, its corresponding *Ywhab* mRNA was downregulated in the same region (Fig. 5G). This discordance strongly suggests that post-transcriptional mechanisms, such as increased translation efficiency or enhanced secretion, contribute to elevated level of YWHAB protein. Additionally, other YWHA family members, such as YWHAZ and YWHAE were also increased in the 3-month entorhinal cortex and CSF of AD patients [54, 55]. Although function of YWHA family in early AD pathogenesis remains limited, accumulating evidence supports their potential as biomarkers for early AD diagnosis.

Finally, ALDOA, a glycolytic enzyme [56], is also known to be elevated in the CSF of early AD patients [53, 57, 58]. We demonstrated that astrocyte-secreted ALDOA was significantly increased in the 3-month entorhinal cortex of AD mice, a change that was absent in the hippocampus at the same time point (Fig. 5H). This finding was consistent with an upregulation of *Aldoa* mRNA in the 3-month entorhinal cortex (Fig. 5I).

#### Clinical relevance of astrocytic secretome

To assess the clinical relevance of astrocytic secretory proteins, we employed SomaScan platform to quantify protein levels in the CSF and jugular venous plasma of　AD　patients and age-matched controls. CSF comprises secretory proteins from multiple neural cell types and holds substantial potential for clinical translation. Comparison with the CSF proteomic data from AD patients identified 36 proteins overlapping with the DEPs from the 3-month entorhinal cortex, of which 55.6% exhibited consistent directional changes (Fig. S9A), including YWHAB and ALDOA. And three were significantly upregulated (Fig. S9B). Notably, PDE1B has been shown to enhance spatial memory when knocked down in animal models and may represent a potential therapeutic target for AD [59, 60]. Proteins in jugular venous plasma are primarily derived from the brain and are less influenced by peripheral tissues. There are 53 overlapping proteins, with 60.4% exhibiting consistent changes (Fig. S9C). Four proteins were significantly upregulated (Fig. S9D). Importantly, HK2, previously associated with increased AD risk in plasma proteomic studies [61], was significantly upregulated in the brains of AD mice and patients, where it impairs microglial phagocytosis and promotes Aβ deposition [62]. These data support the diagnostic and therapeutic potential of early astrocyte-derived DEPs in the entorhinal cortex in AD.

Collectively, these validation experiments underscore the critical role of the entorhinal cortex astrocyte secretome in AD initiation of *APP/PS1* amyloid model. The identified proteins not only highlight key pathogenic pathways, including metabolic disruption, impaired neuronal support, and aberrant signaling, but also confirm their substantial promise as clinically relevant biomarkers during early amyloid deposition stage.

### Lactate dehydrogenase B (LDHB) is an astrocyte-derived neurotoxic factor in early AD

**Fig. 6:**
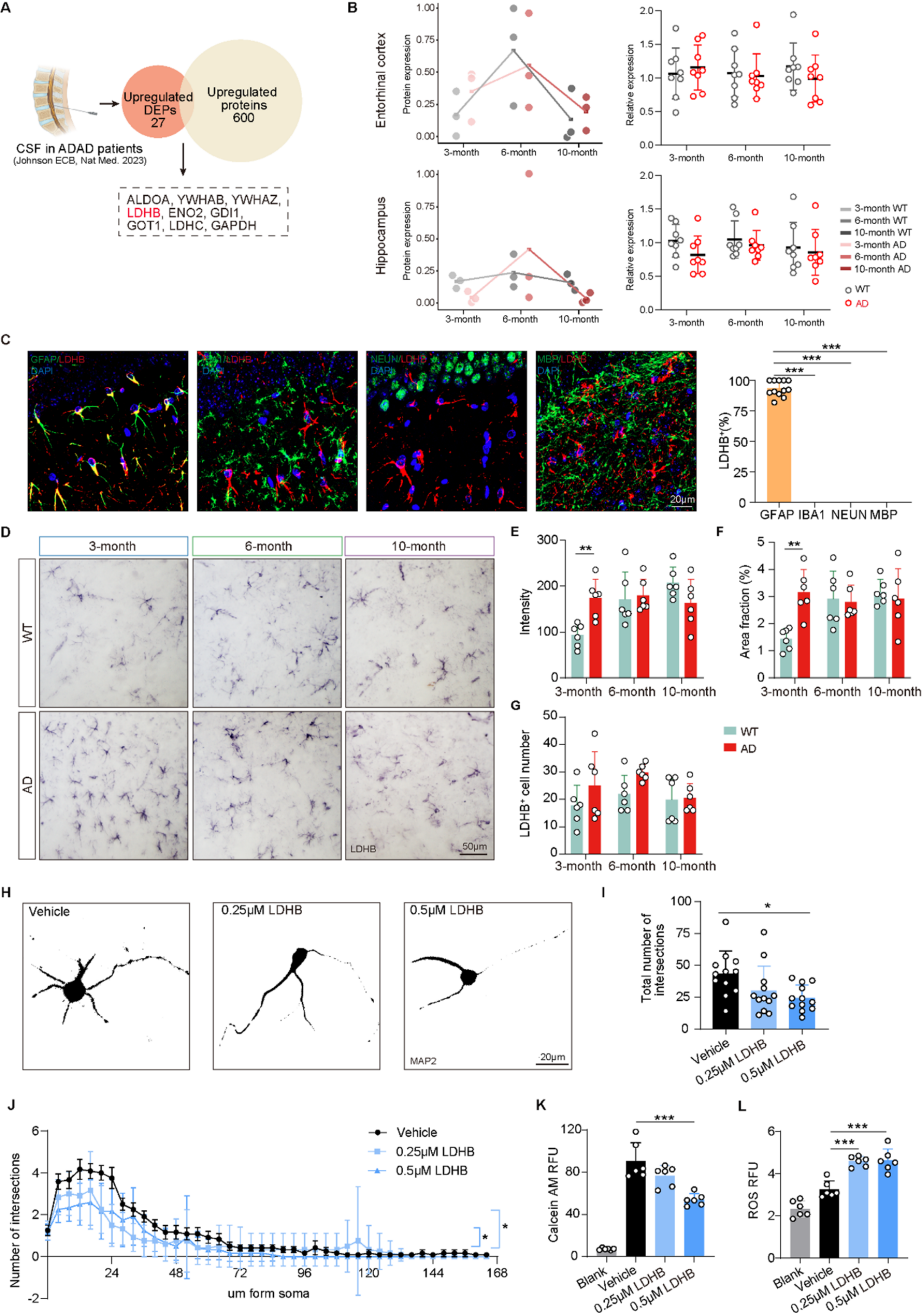
Characterization of the expression pattern and function of LDHB. **A**. Comparison of upregulated proteins in the 3-month entorhinal cortex with DEPs in the CSF of ADAD patients revealed nine overlapping proteins. **B**. In the entorhinal cortex and hippocampus, expression of astrocyte-derived LDHB protein in *APP/PS1* and WT mice across different ages (*n* = 3 biological replicates in mass spectrometry, each replicate was pooled from the entorhinal cortex or hippocampal tissues of 5 mice), and *Ldhb* mRNA expression levels (*n* = 8 mice in RT-qPCR). An unpaired two-tailed Student’s t-test was used for statistical analysis. **C**. Representative images showed that LDHB was specifically highly expressed in astrocyte and exhibited no co-localization with other brain cell types. And expression efficiency of TurboID across astrocytes (GFAP), neurons (NEUN), oligodendrocytes (MBP), and microglia (IBA1) (*n* = 6 mice, 2–3 images per mouse were averaged to yield a single value per mouse). One-way ANOVA tests were used, ***p < 0.001. **D**-**G**. Representative images (**D**) and quantification (E-G) of LDHB expression in the entorhinal cortex (*n* = 6 mice, 2–3 images per mouse were averaged to yield a single value per mouse). An unpaired two-tailed Student’s t-test was used, **p < 0.01. **H**-**J**. Representative images (**H**) and sholl analysis (**I**, **J**) of mouse primary neurons following treatment with varying concentrations of LDHB. Neurons were exposed to LDHB on day 5 of in vitro culture, and morphological assessments were performed on day 10 (*n* = 12 from 3 independent biological replicates. 4 images per replicate, each containing 2–3 neurons, values represent the mean per image). One-way ANOVA tests were used, *p < 0.05. **K**. Cytotoxicity of LDHB measured by Calcein AM staining (*n* = 3 biological replicates, each comprising two duplicate well,). One-way ANOVA tests were used, ***p < 0.001. L. ROS levels induced by LDHB (*n* = 3 biological replicates, each comprising two duplicate well). One-way ANOVA tests were used, ***p < 0.001. DEPs, differentially expressed proteins; CSF, cerebrospinal fluid; ADAD, autosomal-dominant Alzheimer’s disease; WT, wild type; AD, Alzheimer’s disease; RFU; relative fluorescence units; ROS, reactive oxygen species

Among the upregulated proteins in the 3-month entorhinal cortex, we identified Lactate Dehydrogenase B (LDHB) as a top candidate, as it is elevated in the CSF of AD patients up to about 24 years before symptom onset [53, 63] (Fig. 6A). LDHB is a glycolytic enzyme that catalyzes the interconversion of pyruvate and lactate, and generates hydrogen peroxide, inducing oxidative stress [64]. Transcriptomic data confirmed that LDHB was specifically expressed in astrocytes [65]. We confirmed that LDHB is specifically expressed in astrocytes, with an efficiency of ~ 90% (Fig. 6C, S10). In 3-month AD mice, both LDHB protein and its mRNA were upregulated in the entorhinal cortex, whereas were downregulated in the hippocampus (Fig. 6B, D-G), suggesting that entorhinal astrocytes are one of the sources of elevated CSF LDHB in early AD.

To test the functional consequence of this upregulation, we treated primary neurons with exogenous LDHB. We treated neurons with exogenous LDHB at graded concentrations (0.25µM and 0.5µM). At 0.5µM, LDHB induced cytotoxicity, characterized by axonal fragmentation, reduced branching (Fig. 6H-J), neuronal death (Fig. 6K), and increased ROS (Fig. 6L). Concurrently, heat-inactivated LDHB and LDHB-antibody groups were included as controls to validate the functional specificity of LDHB (Fig. S11A, B, C). These findings suggested that during early amyloid deposition, upregulated LDHB in astrocytes of entorhinal cortex was secreted into the extracellular space, where it may exert neurotoxic effects.

## Discussion

This study provides the comprehensive atlas of the astrocyte-specific secretome across distinct brain regions and progressive stages of amyloid pathology. By employing an in vivo TurboID-based proximity labeling strategy, we successfully captured the spatiotemporal dynamics of secretory proteins from astrocytes—a feat unattainable with conventional transcriptomic or whole-tissue proteomic approaches. This methodology establishes a powerful paradigm for dissecting intercellular communication networks in vivo, enabling us to move beyond describing cellular states to directly investigate the molecular dialogues that promote neurodegeneration.

### Astrocyte secretome dysregulation: a bridge between aging and AD pathogenesis

Our findings reveal that even in the early amyloid deposition stage, the astrocyte secretome is profoundly altered, mirroring and likely amplifying age-related cellular decline. The observed reductions in neuroprotective factors (e.g., HOMER1, QDPR), coupled with an upregulation of proteins linked to oxidative stress (e.g., CP, TRNT1), suggest that astrocytes under pathological stress lose their homeostatic functions and adopt a pro-pathogenic profile. When challenged by Aβ pathology, this dysregulation may intensify, creating a vicious cycle where altered astrocytic secretions exacerbate Aβ deposition and fuel neuroinflammation. Consequently, age-related secretome changes may represent a “vulnerability signature” for identifying at-risk individuals, while disease-stage-specific alterations offer a rich reservoir of diagnostic and therapeutic targets.

### The entorhinal cortex as the epicenter of early astrocytic dysfunction

Our data powerfully corroborate the role of the entorhinal cortex as the initial site of AD pathology, demonstrating that profound changes in the astrocyte secretome occur in this region during early amyloid deposition. Notably, the convergent changes observed in our proteomic and transcriptomic datasets revealed that early loss of astrocyte-secreted QDPR in the entorhinal cortex may disrupt neurotransmitter homeostasis and compromise antioxidant capacity [48], while reduced HNRNPAB levels could impair local neuronal morphology and network integrity [51]. Conversely, the early increase of ALDOA, HSPA8 and LDHB reflects enhanced astrocytic secretion, suggesting underlying metabolic dysregulation [56] and formation a neurotoxic microenvironment.

Furthermore, we identified a significant early upregulation of extracellular matrix (ECM) proteins, including HAPLN1 [66] and FBLN5 [67]. These proteins, critical for perineuronal net integrity and synaptic plasticity, were specifically elevated in the 3-month entorhinal cortex. This finding, consistent with human postmortem data [68], suggests that one of the earliest events in *APP/PS1* amyloid model may be an astrocyte-related remodeling of the ECM, creating a microenvironment that is less resilient to subsequent insults and potentially permissive for pathological spread.

### A bidirectional crosstalk between the astrocyte secretome and core AD pathologies

Further analysis revealed a complex, bidirectional interplay between the astrocyte secretome and the amyloid pathologies of AD. We identified 21 secretory proteins for RT-qPCR that overlap with known proteins implicated in AD-related protein aggregation and neurotoxicity [69] (Fig. S12). For instance, the early upregulation of HK2 in the entorhinal cortex astrocytes not only points to metabolic rewiring but also directly implicates astrocytes in promoting Aβ plaque burden, as HK2 loss has been shown to enhance microglial phagocytosis [62, 70]. Conversely, in the late-stage hippocampus, where Aβ pathology is extensive, we observed a significant downregulation of astrocyte-secreted SYP, a key synaptic vesicle protein. This finding aligns with prior evidence demonstrating that Aβ exposure suppressed SYP expression [71]. Our data suggests that toxic protein aggregates impair the capacity of astrocytes to provide synaptic support, thus actively contributing to synaptic failure. This reveals a dynamic dialogue: astrocyte secretions are modulated by Aβ pathology and contribute to the disease process, underscoring their multifaceted role in the pathogenic cascade.

### Reconciling in vivo secretomics with broader omics data

Our study, focused specifically on the astrocyte secretome, provides a crucial layer of cellular specificity to the broader landscape of AD proteomics. Several well-established CSF biomarkers (e.g., SMOC1, primarily from oligodendrocytes) [54, 72] or nuclear proteins (e.g., RORB) [7] were absent from our dataset. This absence is consistent with the design of our experimental strategy, which selectively enriches for astrocyte-derived secretory proteins. It allows us to deconvolve the complex CSF proteome, attributing specific signals to their cellular origins and functional compartments (secretory vs. intracellular). Nevertheless, the absence of certain proteins may also reflect biological particularities, detection limits, enrichment biases, extraction conditions, or database/search constraints inherent to MS data. This work, therefore, serves as a vital reference for interpreting clinical fluid biomarker data and opens new avenues for identifying cell-type specific therapeutic targets.

In this study, we selected GFAP as the astrocytic marker. While GFAP is not a universal marker for all astrocytes, it remains one of the most widely used astrocytic markers in comparable in vivo studies [18, 73]. Its use in our study facilitates consistency with existing literatures. In addition, our study employed the astrocyte-specific gfaABC1D promoter, which is derived from the GFAP promoter, and GFAP co-staining was therefore used to validate that TurboID-mediated labeling originated from astrocytes. Furthermore, given that astrocytes in AD are predominantly in a reactive state, GFAP staining allows preferential assessment of disease-relevant astrocyte populations. ALDH1L1 is a more comprehensive astrocytic marker. GFAP^+^ cells constitute ~ 91% of ALDH1L1^+^ cells (Fig. S13A). TurboID labels ~ 81% of ALDH1L1^+^ cells and ~ 72% of GFAP^+^ cells (Fig. S13B, Fig. 1F), suggesting that our approach also captures a subset of GFAP-low (non-reactive) astrocytes. Future studies incorporating additional markers (e.g., ALDH1L1) or state-specific markers will help to further resolve astrocyte heterogeneity.

The purpose of our study is to investigate the secretory proteins from the astrocytes. ER-TurboID may label proteins from organelles in close proximity to the ER (e.g., contact sites). In this study, we extract extracellular proteins from interstitial fluid using a mild digestion method to avoid contamination by intracellular protein components. Subsequent Western blot analysis of the extracted protein samples revealed no detectable signal for the intracellular protein β-actin (Fig. 1H and Fig. S1D), confirming minimal contamination by intracellular proteins (from ER-proximal organelles) and stored secretory proteins (non-immediate secretion).

The small sample size (*n* = 3) represents a limitation of the present study. In this work, the combination of nominal p values and fold change thresholds was primarily applied as a discovery-oriented strategy to identify candidate DEPs associated with astrocyte secretome alterations. Accordingly, downstream analyses, including GO annotation, pathway enrichment, temporal pattern analysis, and candidate prioritization, should be interpreted within the context of exploratory proteomics. In future studies, we will incorporate larger cohorts of independent biological replicates and further report DEPs following false discovery rate (FDR) correction for multiple testing, thereby strengthening the statistical robustness and translational value of the proteomic analyses. Furthermore, these findings primarily reflect astrocyte secretome dynamics in the context of Aβ pathology and do not capture changes arising from the multi‑pathology landscape of the AD brain. Future studies using models that more fully recapitulate AD pathogenesis are warranted to further validate our observations.

The TurboID⁻/Biotin⁺ technical group, prepared from the hippocampus of 2-month-old wild-type mice, served to identify and subtract the nonspecific binding background inherent to streptavidin enrichment and mass spectrometric detection. As this control defines the basal assay background rather than enabling cross-condition comparisons, additional region-, age-, and genotype-matched TurboID⁻/Biotin⁺ groups were not included. To mitigate nonspecific background, we applied stringent analytical criteria: comparison with the TurboID⁻/Biotin⁺ control, a fold-change cutoff, and subsequent multi-tiered validation (transcriptional, functional, and clinical sample). However, a single such control cannot fully account for background variation across regions, ages, genotypes, or pathological states. Future studies will therefore incorporate matched technical controls to improve both the accuracy and robustness of astrocyte secretome profiling.

In summary, this study charts the dynamic and regionally distinct landscape of the astrocyte secretome in APP/PS1 amyloid model, providing unprecedented insights into the earliest molecular events of the disease. We demonstrate that astrocyte-derived secretory proteins are linked to a cascade of metabolic disruption, ECM remodeling, and synaptic dysfunction in amyloid pathology. Looking forward, these findings lay a critical foundation for the clinical translation of astrocyte-derived biomarkers from CSF or blood for early diagnosis. Furthermore, by illuminating key pathogenic pathways, this secretome atlas provides a treasure trove of novel targets for therapeutic interventions aimed at restoring astrocytic homeostatic functions and severing the vicious cycles that contribute to neurodegeneration.

## Methods

### Animals

Female APPswe/PSEN1dE9 *(APP/PS1)* transgenic mice were obtained from Nanjing MiceBion biotechnology Co., Ltd. (Nanjing). Female C57BL/6 J (wild type, WT) mice were provided by the Animal Centre of Daping Hospital affiliated with the Army Medical University (Third Military Medical University). Female mice were used to exclude the potential influence of sex on AD pathologies in the brain. The transgenic *APP/PS1* mice model carries the human *APP* gene with the Swedish mutant and human presenilin 1 (*PS1*) gene encoding the exon 9 deletion mutation, both under the control of the mouse prion protein (PrP) promoter, which is active in neurons. The model is a classical amyloid pathology mouse model with Aβ deposition as the main pathological feature.

### Establishment of the mouse model

AAV generation: AAV5-gfaABC1D-eGFP-TurboID and the control virus AAV5-gfaABC1D-eGFP were constructed and packaged by Shanghai Taitool bioscience Co., Ltd. (Shanghai). All virus were produced at a titer of 1 × 10^13^ GC/mL.

Stereotaxic injection of AAV: Mice were anesthetized with isofluorane (RWD) using a veterinary vaporizer (RWD). The head was sterilized with 70% ethanol, followed by a midline skin incision. AAV injections were performed using a stereotaxic apparatus according to the following coordinates: anterior–posterior − 2 mm from bregma, medial–lateral ± 1.5 mm, and dorsal–ventral − 1.5 mm (for the hippocampus) or anterior–posterior − 4.0 mm from bregma, medial–lateral ± 3 mm, and dorsal–ventral − 2.1 ~ − 1.5 mm (for the entorhinal cortex) from the brain surface. A total of 4 × 10^9^ GC of virus diluted in 400 nL was injected at each site. After injection, the incision was closed with silk sutures. Mice were allowed to recover in a heated cage for 1 h before being returned to their home cages.

Biotin labeling: After a 4-week period to allow viral expression, 1 µL of biotin (5 mg/mL, Sigma) or PBS was injected in situ once daily for 5 consecutive days to achieve sufficient biotin labeling.

In the pilot study in Fig. 1, viral vectors were stereotaxically injected into the hippocampus of 2-month-old WT mice. TurboID^+^/Biotin^+^ group: AAV5-gfaABC1D-eGFP-TurboID expression followed by biotin administration; TurboID^+^/Biotin^−^ group: AAV5-gfaABC1D-eGFP-TurboID expression followed by PBS administration; TurboID^−^/Biotin^+^ group: AAV5-gfaABC1D-eGFP expression followed by biotin administration; TurboID^−^/Biotin^−^ group: AAV5-gfaABC1D-eGFP expression followed by PBS administration.

In the formal experiment in Fig. 2, it was also divided into four groups, and the administration protocol was consistent with that described above. In TurboID^+^/Biotin^+^ experimental group, AAV5-gfaABC1D-eGFP-TurboID was stereotaxically injected into the entorhinal cortex or hippocampus of WT mice and *APP/PS1* mice at 2, 5, and 9 months of age, followed by biotin administration. TurboID^−^/Biotin^+^ group was used as technical control to exclude nonspecific proteins, in which AAV5-gfaABC1D-eGFP was stereotactically injected into the entorhinal cortex or hippocampus of 2-month-old WT mice, followed by biotin administration. TurboID^+^/Biotin^−^ and TurboID^−^/Biotin^−^ control groups were established to validate the functional and regional specificity of TurboID-mediated biotinylation. In TurboID^+^/Biotin^−^ group, AAV5-gfaABC1D-eGFP-TurboID was stereotaxically injected into the entorhinal cortex or hippocampus of 2-month-old WT mice, followed by PBS administration. In TurboID^−^/Biotin^−^ group, AAV5-gfaABC1D-eGFP was stereotaxically injected into the entorhinal cortex or hippocampus of 2-month-old WT mice, followed by PBS administration.

### Extraction of secretory proteins

One day after the final biotin administration, brain tissues (cerebellum, prefrontal cortex, hippocampus, and entorhinal cortex) were dissected and collected. Tissues pooled from 5 mice were combined into one Eppendorf tube to generate one biological replicate. Samples were kept on ice to prevent proteins degradation. Each sample was weighed, and type III collagenase (Worthington, 75 U/mL) in Hibernate-E buffer (Thermo Fisher Scientific) was added (800 µL per 100 mg of tissue). The tissues were cut into 2–3 mm³ pieces using sterile scissors and incubated at 37 °C for 20 min, with gentle mixing every 5 min. Following incubation, protease and phosphatase inhibitors (Roche) were added. Samples were then centrifuged at 300 × g for 5 min at 4 °C. The supernatant was transferred to a new tube and centrifuged at 12,000 × g for 30 min at 4 °C to collect total secretory proteins, which could be directly used for mass spectrometry analysis of total secretory proteins.

### Extraction of intracellular proteins

RIPA lysis buffer containing protease and phosphatase inhibitors was added to the pellet obtained in the previous step, and the samples were incubated on ice for 10 min. Samples were then briefly vortexed, followed by three rounds of 10 s sonication using a Sonics VCX130 sonicator. The lysates were subsequently centrifuged at 12,000 × g for 10 min at 4 °C to collect intracellular proteins.

### Enrichment of biotinylated secretory proteins

200 µL Dynabeads™ MyOne™ Streptavidin T1 magnetic beads (Thermo Fisher Scientific) were used for the enrichment of biotinylated secretory proteins each sample. Briefly, the beads were washed three times with 1 mL washing buffer (PBS + 1% NP-40(Sigma)). Extracted total secretory proteins (500 µL) were then added and incubated with the breads overnight at 4 °C with rotation. After incubation, the beads were washed three times with 1 mL washing buffer. Subsequently, 60 µL 2× loading buffer (Solarbio) with 20 mM DTT (Sigma) and 2 mM biotin was added, and the samples were boiled at 95 °C for 10 min to elute the biotinylated proteins bound to the beads. The eluted proteins were then subjected to SDS-PAGE, and the corresponding gel bands were excised and submitted for silver staining and mass spectrometry analysis.

### Silver staining

The gel was stained using a fast silver stain kit (Beyotime). Briefly, the gel was fixed in a fixative solution, including 50% ethanol (Chuandong chemical) and 10% acetic acid (Kelong chemical), for 20 min, washed with 30% ethanol for 10 min, and then rinsed with water for 10 min. The gel was incubated with 1× silver staining sensitizing solution for 2 min, washed twice with water, incubated with 1× silver staining solution for 10 min, and washed with water. Finally, the gel was incubated with developing solution (1× silver staining basic developing solution and developing accelerator) until clear positive bands appeared, at which point the developing reaction was terminated.

### LC-MS/MS data acquisition

#### For gel bands

Proteins extraction and digestion: The target gel bands were cut into small gel pieces and transferred into 1.5 mL Eppendorf tubes. 1 mL of 50% acetonitrile (Thermo Fisher Scientific) was added and incubated; the liquid was then discarded. This process was repeated until the gel particles became colorless. 1 mL of acetonitrile was added, and after incubation until the gel pieces turned white and viscous, the samples were vacuum-concentrated to dryness for 10 min. 100 µL of 20 mM tris(2-carboxyethyl) phosphine (Adamas) and 1 mL of acetonitrile were added, followed by incubation until the gel pieces appeared white and viscous. The samples were again vacuum-concentrated to dryness. 100 µL of 50 mM iodoacetamide (Sigma) was added, along with 1 mL of acetonitrile. After incubation until the gel pieces turned white and viscous, the samples were vacuum-concentrated to dryness. Subsequently, 100 µL of 10 ng/µL trypsin solution (Hualishi Tech) was added, and digestion was performed overnight at 37℃. 100 µL of 5% formic acid (Thermo Fisher Scientific) in 50% acetonitrile was added, and the liquid was collected into a 1.5 mL Eppendorf tube. 100 µL of 70% acetonitrile was added, and the liquid was collected into the same 1.5 mL Eppendorf tube. 100 µL of 100% acetonitrile was added, and after incubation until the gel pieces became white and viscous, the liquid was collected into the 1.5 mL Eppendorf tube. The samples were vacuum-concentrated to dryness. After desalting, the samples were centrifugally concentrated to dryness at 40℃ under conditions below 10 mBar. Following reconstitution, the peptide concentration was measured at a wavelength of 280 nm.

LC-MS/MS data acquisition: The resulting peptides (400 ng) were analyzed by LC–MS/MS. LC-MS/MS analysis was performed using a Vanquish Neo UHPLC system and an Orbitrap Exploris 480 mass spectrometer (Thermo Fisher Scientific) equipped with a FAIMS Pro™ (Thermo Fisher Scientific) interface, employing data-independent acquisition (DIA) for data collection; data-dependent acquisition (DDA) was used for library construction. Mobile phase A consisted of 2% acetonitrile (Thermo Fisher Scientific), 98% water, and 0.1% formic acid (Thermo Fisher Scientific); mobile phase B consisted of 20% water, 80% acetonitrile, and 0.1% formic acid. All reagents were of mass spectrometry grade. For all samples, peptides were loaded onto a pre-column (3 μm, 100 Å, 20 mm × 75 μm i.d.) at a flow rate of 6 µL/min, then eluted onto an analytical column (1.9 μm, 120 Å, 150 mm × 75 μm i.d.) at a flow rate of 300 nL/min, with analysis performed using a 30-minute liquid chromatography gradient (8% to 35% mobile phase B).The MS scanning parameters for Part 1 were as follows: FAIMS CV (Compensation Voltage) was set to -65 V and − 45 V. The full scan range was 390–1010 m/z, with a resolution of 60,000 (at 200 m/z), a normalized AGC target of 300%, and a maximum ion injection time (max IT) of 50 ms. The MS/MS resolution was 30,000, with a normalized AGC target of 2000%, a standard collision energy of 32%, and a maximum ion injection time (max IT) of 54 ms; for FAIMS CV -65 V, the parent ion mass range was 400–520 m/z, with an isolation window of 10 m/z, a window overlap of 1 m/z, and 12 windows; for FAIMS CV -45 V, the parent ion mass range was 630–750 m/z, with an isolation window of 10 m/z, a window overlap of 1 m/z, and 12 windows. The MS scanning parameters for Part 2 were as follows: FAIMS CV (Compensation Voltage) was set to -65 V and − 45 V. The full scan range was 390–1010 m/z, with a resolution of 60,000 (at 200 m/z), a normalized AGC target of 300%, and a maximum ion injection time (max IT) of 50 ms. The MS/MS resolution was 30,000, with a normalized AGC target of 2000%, a standard collision energy of 32%, and a maximum ion injection time (max IT) of 54 ms; for FAIMS CV -65 V, the parent ion mass range was 520–630 m/z, with an isolation window of 10 m/z, a window overlap of 1 m/z, and 11 windows; for FAIMS CV -45 V, the parent ion mass range was 750–1010 m/z, with an isolation window of 20 m/z, a window overlap of 1 m/z, and 13 windows.

Database search: Raw data were searched using DIA-NN (v1.8.1) in MBR mode. The FASTA sequence file employed was 2023-06-19-reviewed-contam-UP000000589_PD_mouse.fasta (downloaded from UniProt, containing 17,268 reviewed proteins and contaminant proteins). Fixed modifications were set to carbamidomethylation of cysteine (+ 57.021464 Da) and protein N-terminal methionine excision (-131.040485 Da). Variable modifications were set to oxidation of methionine (+ 15.994915 Da) and TurboID-mediated biotinylation of lysine (+ 226.077598 Da). The parent ion false discovery rate (FDR) was set to 0.01.

#### For total secretory proteins

Proteins extraction and digestion: Total secretory proteins were transferred to a PCT™ MicroTube, followed by sequential addition of 30 µL lysis buffer (6 M urea (Sigma), 2 M thiourea (Sigma), 5 µL 0.2 M tris(2-carboxyethyl) phosphine (Adamas), 2.5 µL 0.8 mM iodoacetamide, and 12.5 µL 0.1 M ammonium bicarbonate (Greagent)). After sealing, the tube was placed in a Barocycler (Pressure BioSciences) and processed for 90 cycles at 45 k psi (30 s high pressure/10 s atmospheric pressure, 30℃). Subsequently, 85 µL 0.1 M NH4HCO3 buffer (Greagent), 10 µL trypsin (enzyme-to-protein ratio 1:20, w/w) and 5 µL recombinant Lys-C (1:80, w/w) (Hualishi Tech) were added. Enzymatic digestion was performed in the Barocycler for 120 cycles at 20 k psi (50 s high pressure/10 s atmospheric pressure, 30℃). Reactions were quenched with 15 µL 10% (v/v) trifluoroacetic acid (Thermo Fisher Scientific). Peptides were desalted on a SOLAµ SPE 96-well plate (Thermo Fisher Scientific), dried and re-dissolved. Peptide concentration was determined at 280 nm.

LC-MS/MS data acquisition: Peptides (200 ng) were loaded at 800 bar onto a trap column (5 μm, 5 mm × 300 μm i.d.) using a Vanquish Neo UHPLC coupled to an Orbitrap Astral mass spectrometer (Thermo Fisher Scientific). Peptide separation was carried out on an analytical column (1.9 μm, 120 Å, 150 mm × 75 μm i.d.) at 500 nL min⁻¹ with a 19.5 min gradient: 0–0.5 min, 8% B; 0.5–1.5 min, 8–10% B; 1.5–17.5 min, 10–30% B; 17.5–19.5 min, 30–40% B (mobile phase A: 2% acetonitrile (Thermo Fisher Scientific), 0.1% formic acid (Thermo Fisher Scientific) in water; mobile phase B: 80% acetonitrile, 0.1% formic acid in water). DIA was performed with FAIMS compensation voltage − 42 V. MS¹ spectra were acquired from m/z 380–980 using 299 isolation windows (2 m/z each, 0 m/z overlap) at 240,000 resolution (m/z 200), AGC target 500% and 3 ms maximum injection time. MS² spectra were recorded from m/z 150–2000 at 500% AGC target, 25% normalized collision energy and 3 ms maximum injection time.

Database search: Raw data were searched using DIA-NN (v1.8.1) in MBR mode. The FASTA sequence file employed was 2023-06-19-reviewed-contam-UP000000589_PD_mouse.fasta. Fixed modification was set to carbamidomethylation of cysteine (+ 57.021464 Da). Variable modification was set to oxidation of methionine (+ 15.994915 Da). The parent ion false discovery rate (FDR) was set to 0.01.

### Proteomics data analysis

For LC‑MS/MS analysis, three biological replicates were prepared per group, with each replicate consisting of pooled entorhinal cortex or hippocampal tissue from five mice, resulting in an effective sample size of *n* = 3 independent pools per condition. All statistical and bioinformatics analyses were performed in R (version R 4.3.3/4.5.0).

#### Screening of astrocyte-derived secretory proteins

Astrocyte-derived secretory proteins were screened by comparing protein expression levels between each experimental groups and the technical control group (TurboID^+^/Biotin^+^ vs. TurboID^−^/Biotin^+^, *p* < 0.05 and log_2_FC > 1). The proteins list is provided in Supplementary Table 3.

#### Comparison of secretory proteins from astrocytes and total cells

For the entorhinal cortex and hippocampus regions, astrocyte-specific secretory proteins were identified separately in *APP/PS1* mice and WT mice across different ages. Proteins were considered astrocyte-specific if they met two criteria: (1) detection in at least one biological replicate (non-zero value), and (2) exclusive expression in astrocytes relative to other cell types. Scatter plots of iBAQ values were generated to visualize the intensity distribution of these astrocyte-specific secretory proteins against all detected secretory proteins in astrocytes under each experimental condition.

#### Proteins trajectory analysis

Secretory proteins in WT were clustered into 9 clusters based on their mean expression trends across 3-, 6-, and 10-month time-points. LOESS regression was implemented using the R stats package to calculate predicted expression values, with resulting curves visualized via the ggplot2 package. To make it easier to classify and compare the trend changes of proteins, expression values for each protein were min-max normalized to a [0,1] scale. Trend change classifications were subsequently defined through quantitative assessment of: (1) slopes between temporally adjacent nodes, and (2) differential expression magnitudes at nodal time-points.

#### DEPs analysis during aging

The distribution parameters of the proteins data matrix were estimated using the MASS package, with missing values imputed by the minimum value derived from the fitted distribution. Differential expression analysis of secretory proteins across different ages was performed using Student’s t-test implemented in the R stats package, with subsequent calculation of FC values. Significant proteins (*p* < 0.05) were then subjected to GO enrichment analysis, and we visualized differential expression patterns and enriched GO profiles in the entorhinal cortex and hippocampus across the 3- to 6-month and 6- to 10-month periods.

#### Screening of DEPs in AD

DEPs were identified by performing Student’s t-test and calculating FC values (*p* < 0.05 and |log_2_FC|>1) using the stats package in R. Additionally, the results illustrated DEPs in the entorhinal cortex and hippocampus across different ages, and significant pathways enriched through GO analysis. The DEPs list is provided in Supplementary Table 3.

#### PCA analysis

Principal component analysis (PCA) was performed using the stats package in R, and separation patterns among experimental groups were visualized via the ggplot2 package.

### Immunohistochemistry and immunofluorescence

The left hemi-brain of each mouse was sectioned coronally or sagittally at 20 μm using a freezing sliding microtome. For immunohistochemistry, two coronal sections corresponding approximately to bregma coordinates − 2.1, and − 3.0 m, were stained with LDHB (1:200, Invitrogen). For immunofluorescence, two coronal sections − 2.1 and − 3.0 mm and three sagittal sections + 2.7, + 2.9, + 3.1 mm were stained with LDHB (1:200, Invitrogen), GFAP (1:1000, Abcam), NEUN (1:1000, Abcam), IBA1 (1:1000, Invitrogen), MBP (1:500, Sigma), Biotin (1:1000, Abcam) and ALDH1L1 (1:100, Cell Signaling Technology).

### Western blot

Proteins samples were loaded on a 4 to 20% SDS–polyacrylamide gel electrophoresis, and then transferred onto nitrocellulose (NC) membranes. After blocking with 5% fat-free milk for 1 h, the NC membranes were incubated with the following primary antibodies overnight at 4 °C: anti-V5 antibody (1:1000, Invitrogen) to detect TurboID; anti-Actin antibody (1:1000, Cell Signaling Technology) to detect Actin; anti-Biotin antibody (1:1000, Abcam) to detect biotin. The membranes were incubated with the corresponding IRDye 800 CW-conjugated secondary antibodies (1:10000, LI-COR) and scanned using an Odyssey fluorescent scanner. Relative band intensities were normalized to the band intensity of the internal reference proteins for analysis.

### Reverse-transcription quantitative PCR (RT-qPCR) assays

3-, 6- and 10-month-old *APP/PS1* and WT mice were selected for detection, with 8 mice in each group. The entorhinal cortex and hippocampus tissues were isolated to extracte total RNA for RT-qPCR analysis. The results were expressed as the relative mRNA expression level (log_2_FC) of *APP/PS1* mice compared with age-matched WT mice. The brain tissues were homogenized in Trizol reagent (Thermo Fisher Scientific). RNA was extracted by using Trizol reagent followed by quantification with NanoDrop ND-100 spectrophotometer (Thermo Fisher Scientific). cDNA was synthesized by reverse transcription for qPCR with Hifair^®^ III 1st Strand cDNA Synthesis SuperMix for qPCR (Yeason) on CFX96 real-time PCR system (Bio-Rad). RT-qPCR was performed using Hieff UNICON^®^ Universal Blue qPCR SYBR Green Master Mix (Yeason) on CFX96 Real-Time PCR Detection System (Bio-Rad). Glyceraldehyde-3-phosphate dehydrogenase (*Gapdh*) served as the internal control. The sequences of RT-qPCR primers are listed in Supplementary Table 1.

### Isolation of mouse primary neurons

The plates were seeded and the corresponding culture mediums were prepared one day in advance. Seeding solution: Boric acid sodium buffer (Macklin) containing 10 ug/mL poly-D-lysine (Sigma) and 5 ug/mL laminin (Gibco). Plating medium: Neurobasal medium (Gibco) with 1% B27 (Gibco), 1% penicillin/streptomycin (P/S, Gibco), 1% sodium pyruvate (Gibco), 10% fetal bovine serum (Gibco), and 1% glutamax (Gibco). Maintenance medium: Neurobasal medium (Gibco) with 1% B27 (Gibco), 1% P/S (Gibco), 1% sodium pyruvate (Gibco), and 1% glutamax (Gibco).

Primary neurons were isolated from embryonic day 16 mice. Following the collection of mice embryos and brains, the cerebral cortex and hippocampus were dissected and the meninges were carefully removed. The tissues were minced with fine scissors and enzymatically digested with papain (Sangon Biotech) and DNase I (Stemcell). The digestion was terminated with plating medium, and the cell suspension was centrifuged at 1500 rpm for 8 min. The supernatant was discarded, and the cell pellet was resuspended in plating medium. After cell counting, neurons were seeded at different densities: 2 × 10⁵ cells per well in 24-well plates (with coverslips), and 3 × 10⁴ cells per well in 96-well plates. The plating medium was replaced with maintenance medium 3–6 h after seeding. The maintenance medium was refreshed every other day thereafter.

### Cytotoxicity and ROS assays of LDHB

Five days after isolation, when neuronal neurites were fully extended, neurons were treated with varying concentrations (0.25µM, 0.5µM) of LDHB (MedChemExpress). After 5 days of treatment, MAP2 immunofluorescence staining (1:1000, Invitrogen) was performed on coverslips to assess neuronal morphology. Concurrently, cytotoxicity was evaluated using a Calcein AM assay kit (Beyotime), and ROS level was evaluated by a Reactive Oxygen Species assay kit (Beyotime). In addition, a 0.05µM LDHB inhibitor AXKO-0046 (MedChemExpress) group, a 0.5µM LDHB + 1µM antibody (Invitrogen) group, a 1µM antibody group, and a 0.5µM LDHB heat inactivation group (95℃, 10 min) were added as controls. The same method was used for detection. The recombinant LDHB protein (MedChemExpress) was subjected to rigorous endotoxin removal and tested by the LAL assay, yielding an endotoxin level below the detection limit, thereby excluding endotoxin interference in the cytotoxicity experiments. All groups were treated with identical buffer systems to rule out solvent effects. The results of the LDHB in vitro functional assays were derived from three independent biological replicates, each performed using primary neurons isolated from separate batches rather than as technical replicates or replicate wells from the same cell preparation. The data shown represent the combined analysis of these three independent experiments, thereby ensuring reproducibility and statistical reliability.

### Cytotoxicity assays of HSPA8

When neuronal neurites were fully extended, neurons were treated with varying concentrations (0.05µM, 0.25µM and 0.5µM) of HSPA8 (MedChemExpress). After 1 days of treatment, MAP2 immunofluorescence staining (1:1000, Invitrogen) was performed on coverslips to assess neuronal morphology. Concurrently, cytotoxicity was evaluated by a Calcein AM assay kit (Beyotime) and an enhanced cell counting kit-8 (Beyotime).

### Cytotoxicity assays of QDPR

Three days after isolation, neurons were treated with varying concentrations (0.05µM, 0.25µM and 0.5µM) of QDPR (MedChemExpress). After 5 days of treatment, MAP2 immunofluorescence staining (1:1000, Invitrogen) was performed on coverslips to assess neuronal morphology. Concurrently, Cell supernatant was evaluated by a dopamine assay kit (Beyotime), and cell was evaluated by a Reactive Oxygen Species assay kit (Beyotime) and a Calcein AM assay kit (Beyotime).

### SomaScan proteomics data processing

Jugular venous plasma and cerebrospinal fluid samples were obtained from AD patients and cognitively normal controls previously recruited from the Department of Neurology, Daping Hospital, Army Medical University (Third Military Medical University). The study was approved by the Institutional Review Board of Daping Hospital, and informed consent was obtained from all participants and their caregivers.

Plasma samples from 10 AD patients and 10 age-matched cognitively normal controls, and CSF samples from 8 AD patients and 10 age-matched cognitively normal controls were included for proteomic analysis. Detailed clinical information is provided in Supplementary Table 2. Plasma proteomics was performed using the SomaScan 11 K Assay v5.0 (SomaLogic), which quantifies ~ 11,000 protein measurements covering over 10,000 unique human proteins from small-volume samples. All experimental procedures were conducted by Accuramed Technology Limited. Protein abundance was reported as relative fluorescence units (RFU). Preprocessed and normalized data (SQS reports and ADAT files) were generated using SomaLogic’s DataDelve platform. To minimize technical variation, raw RFU data were subjected to a standardized normalization workflow, including hybridization normalization, intra-plate median signal normalization, plate scaling and calibration, adaptive normalization by maximum likelihood, and final quality control assessment.

For differential expression analysis, FC was calculated from group medians of RFU values, and Welch’s t-test was applied to log10-transformed RFU data, with *p* < 0.05 as the significance threshold. Expression patterns were visualized using a heatmap generated with pheatmap package (version 1.0.12).

## Electronic Supplementary Material

Below is the link to the electronic supplementary material.

Supplementary Material 1

Supplementary Material 2

Supplementary Material 3

Supplementary Material 4

Supplementary Material 5

Supplementary Material 6

Supplementary Material 7

Supplementary Material 8

Supplementary Material 9

Supplementary Material 10

Supplementary Material 11

Supplementary Material 12

Supplementary Material 13

Supplementary Material 14

Supplementary Material 15

Supplementary Material 16

## Data Availability

The mass spectrometry proteomics data have been deposited to the ProteomeXchange Consortium (https://proteomecentral.proteomexchange.org) via the iProX partner repository [74, 75] with the dataset identifier PXD078262 and PXD078308. All bioinformatics analysis scripts are publicly available in the Zenodo repository at 10.5281/zenodo.20131790.
