# Supplementary figures and images for: In vivo profiling of astrocyte secretome reveals brain-region specific regulatory networks in a mouse model of amyloid pathology
Source: Mol Neurodegener. 2026 May 23;21:39. doi: 10.1186/s13024-026-00956-y (PMC13393950; doi:10.1186/s13024-026-00956-y)

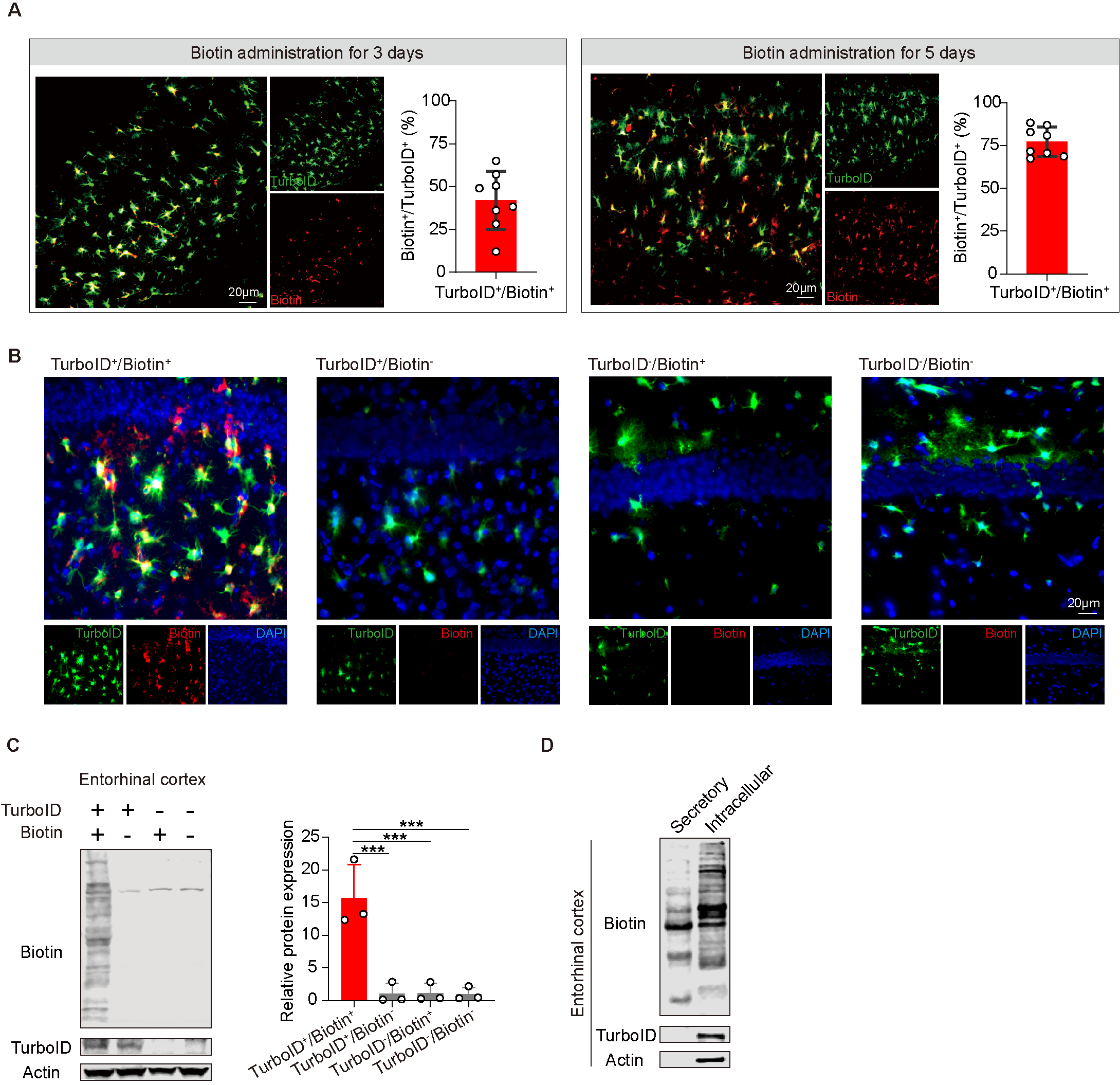

Supplement: Supplementary file 1 — Supplementary Material 1 [file 13024_2026_956_MOESM1_ESM.tif]

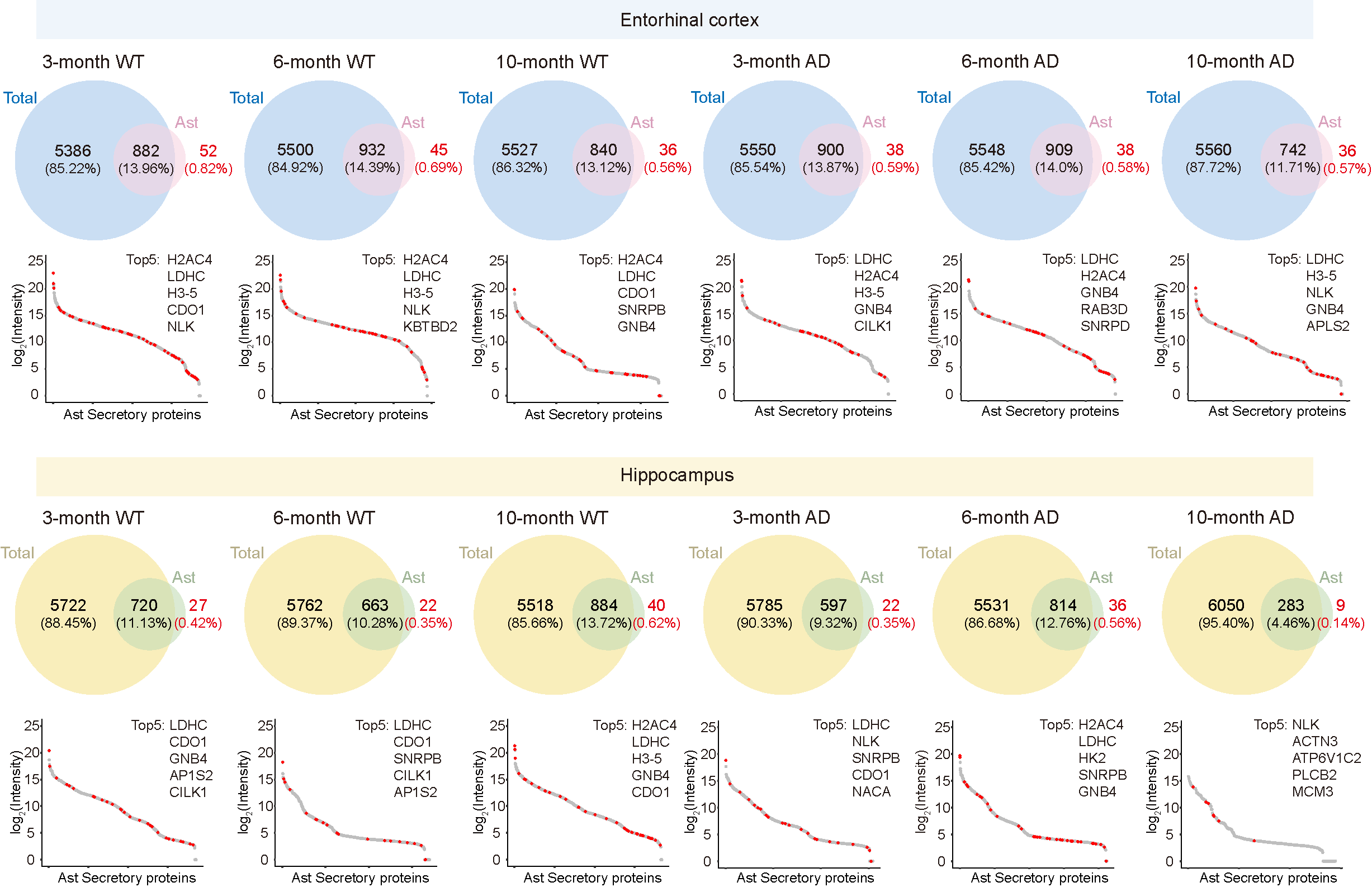

Supplement: Supplementary file 2 — Supplementary Material 2 [file 13024_2026_956_MOESM2_ESM.tif]

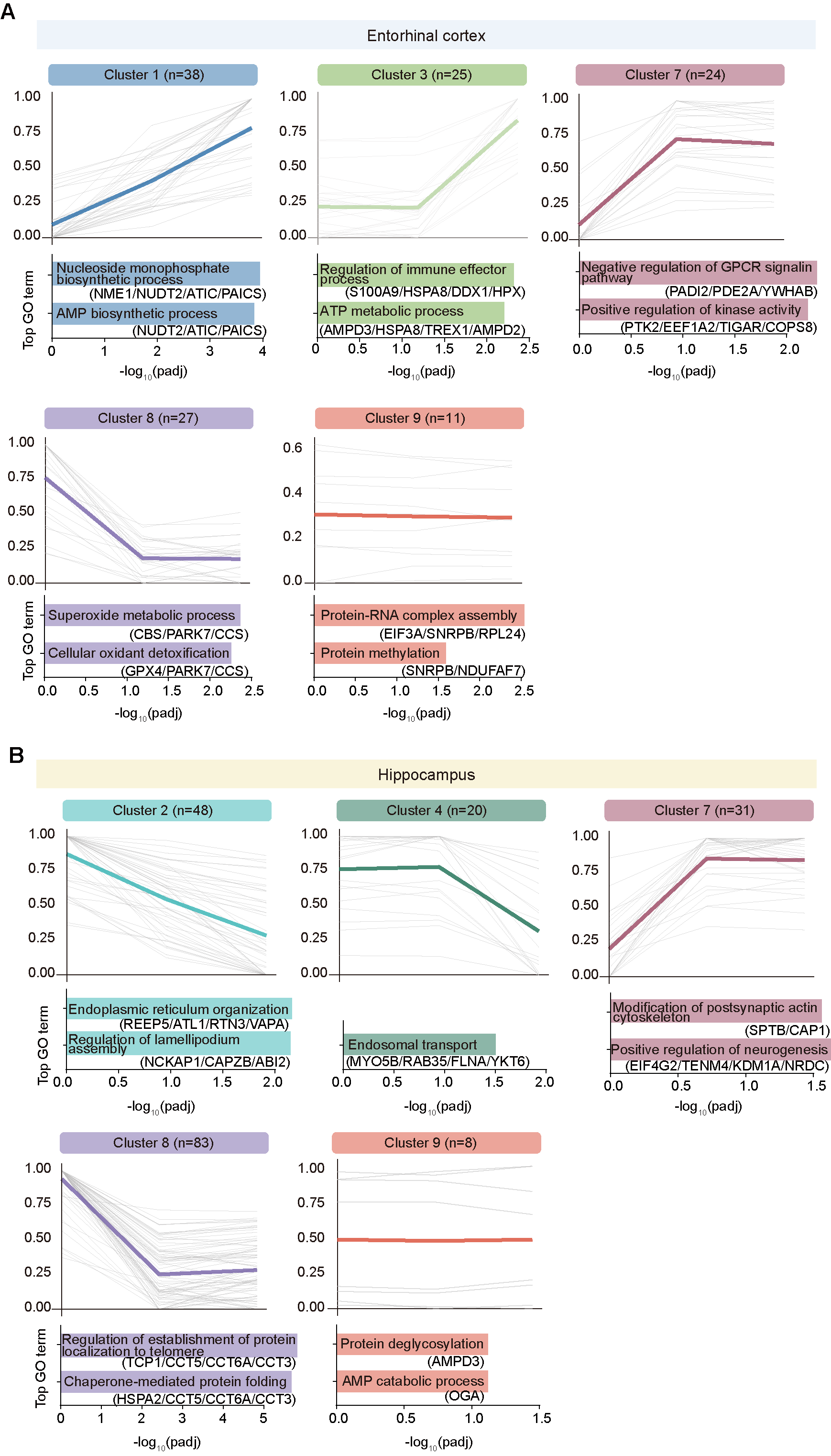

Supplement: Supplementary file 3 — Supplementary Material 3 [file 13024_2026_956_MOESM3_ESM.tif]

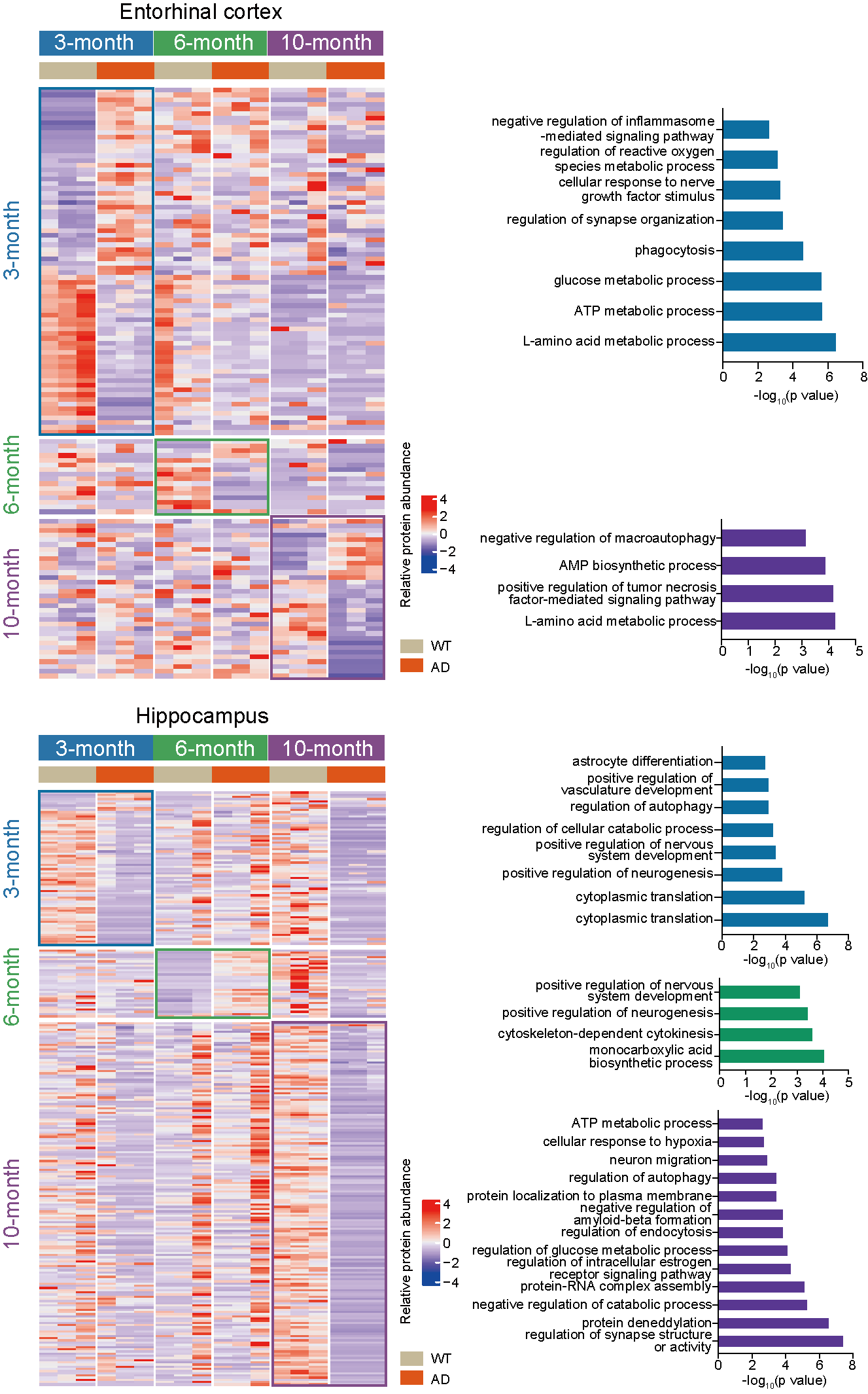

Supplement: Supplementary file 4 — Supplementary Material 4 [file 13024_2026_956_MOESM4_ESM.tif]

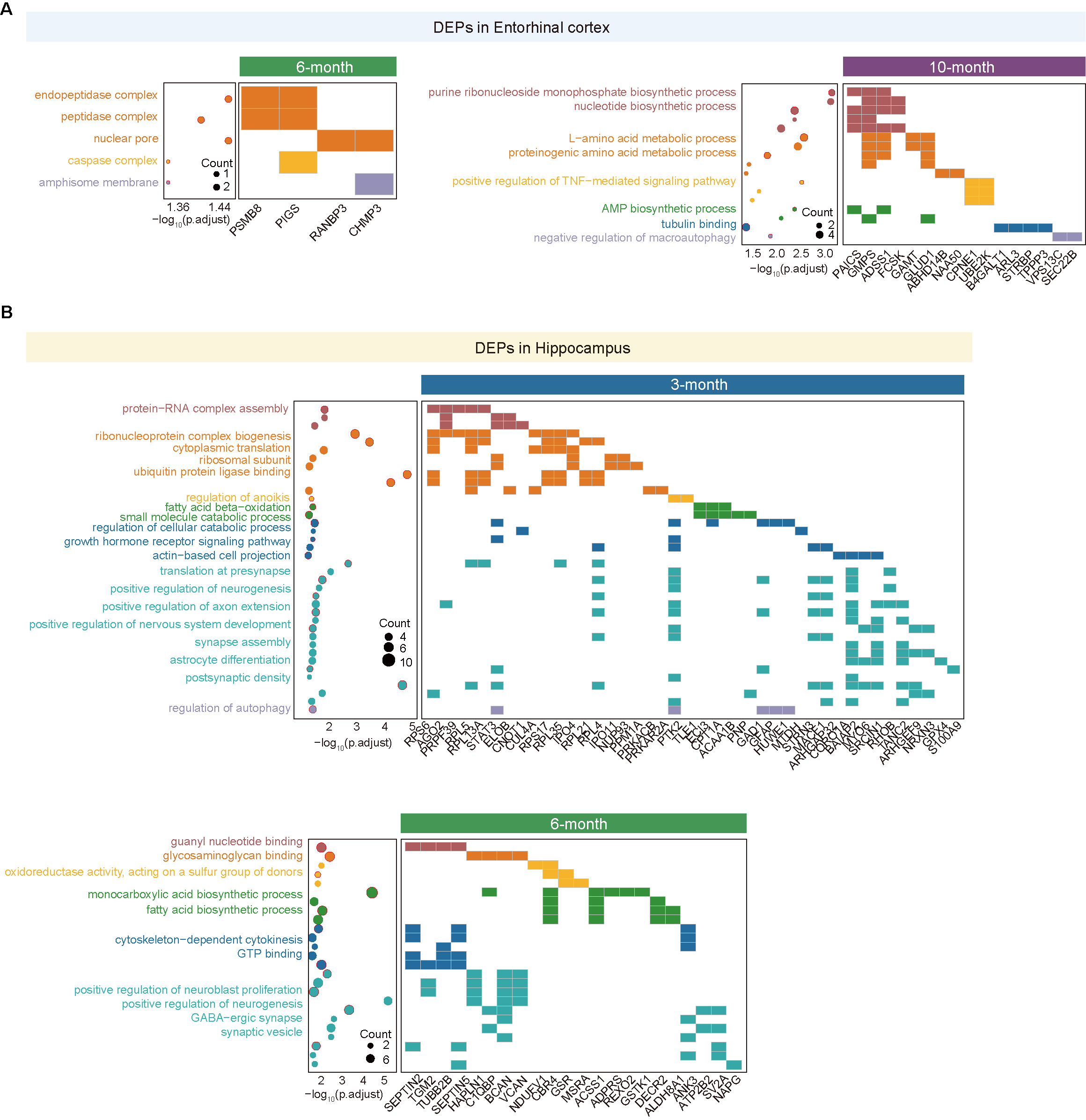

Supplement: Supplementary file 5 — Supplementary Material 5 [file 13024_2026_956_MOESM5_ESM.tif]

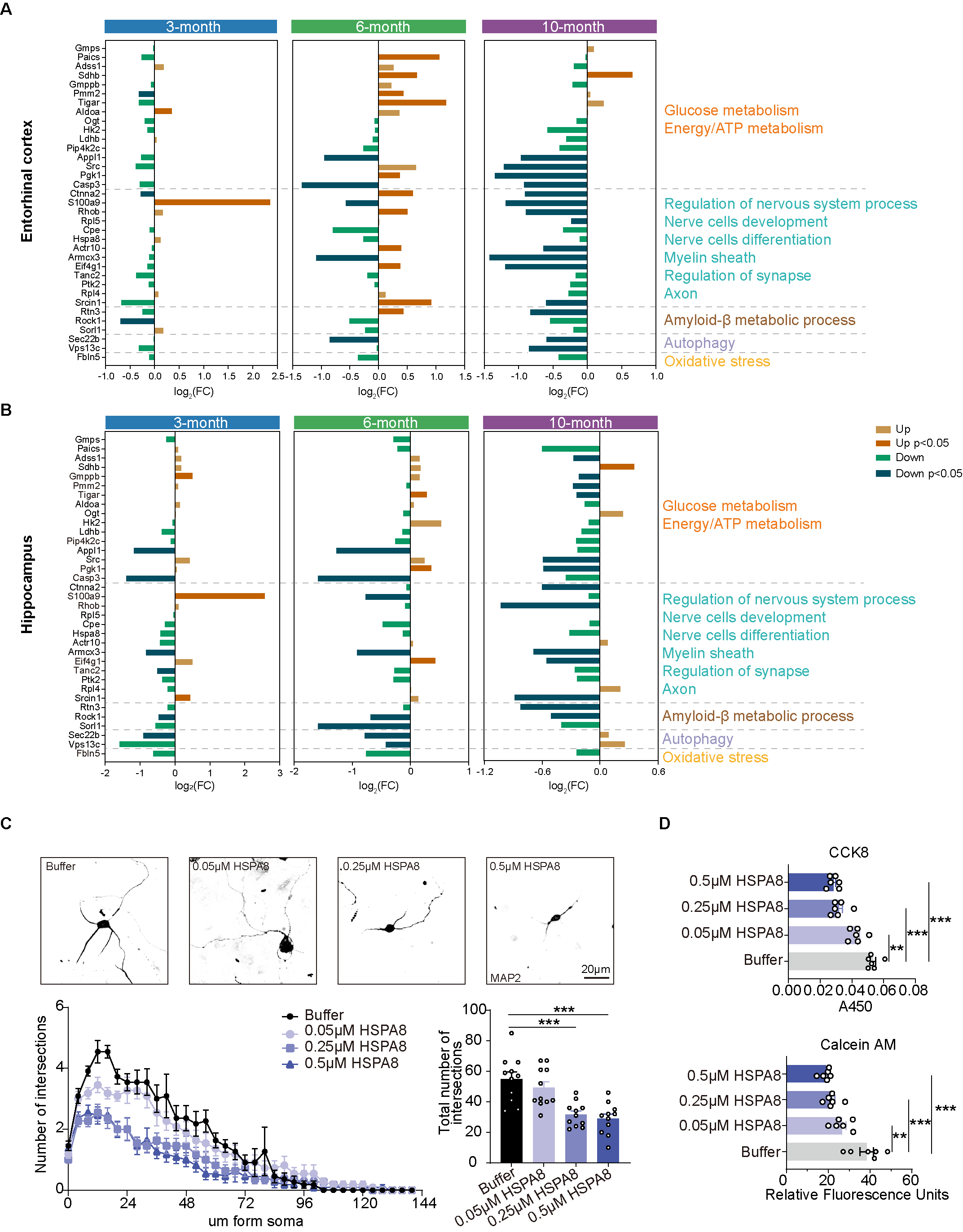

Supplement: Supplementary file 6 — Supplementary Material 6 [file 13024_2026_956_MOESM6_ESM.tif]

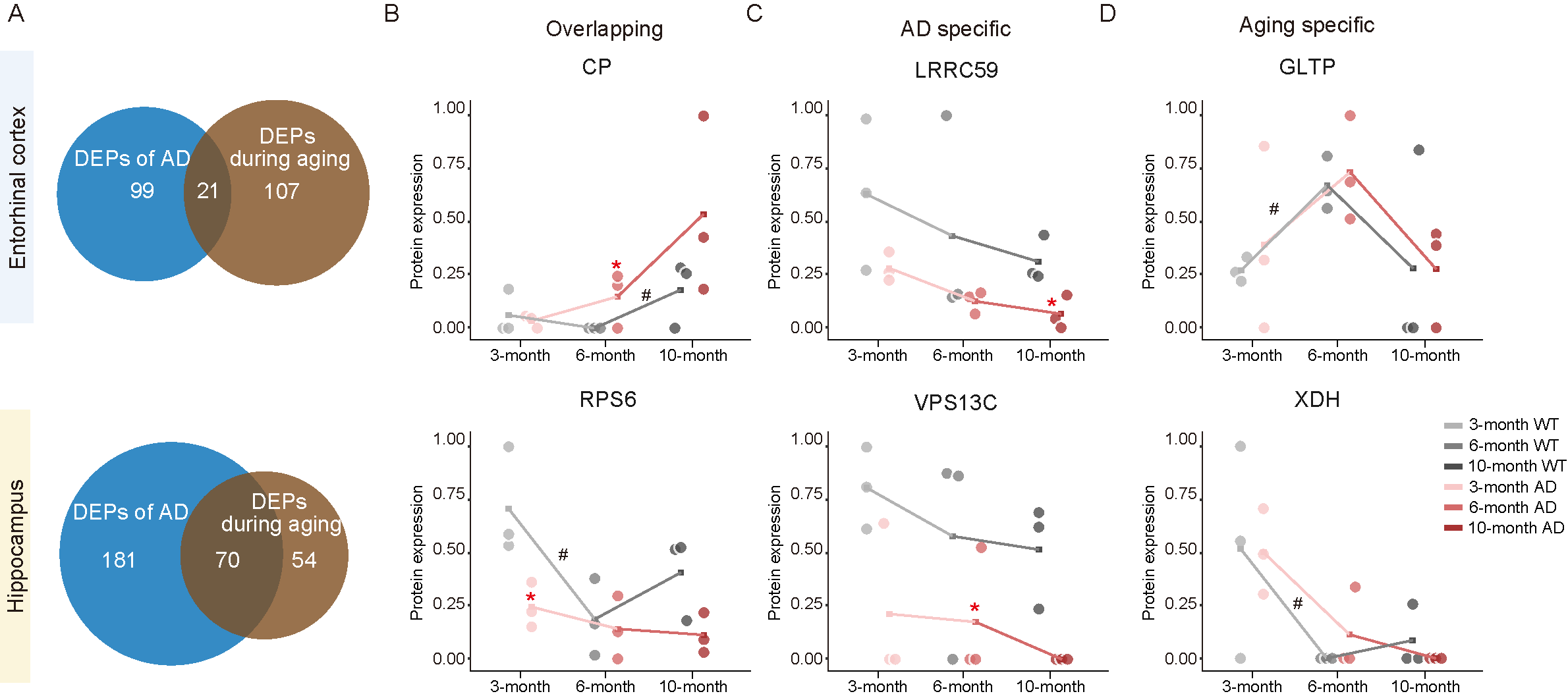

Supplement: Supplementary file 7 — Supplementary Material 7 [file 13024_2026_956_MOESM7_ESM.tif]

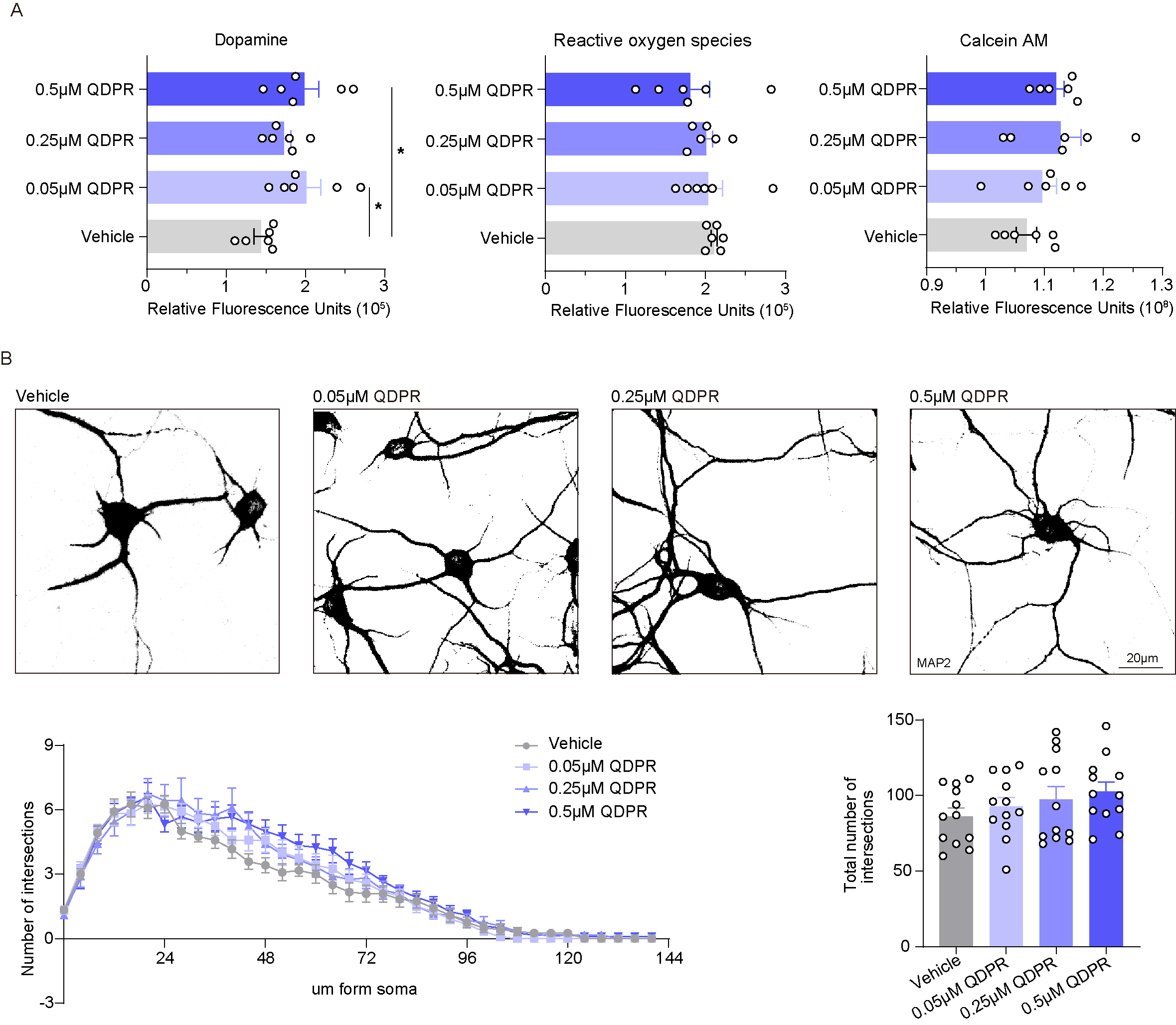

Supplement: Supplementary file 8 — Supplementary Material 8 [file 13024_2026_956_MOESM8_ESM.tif]

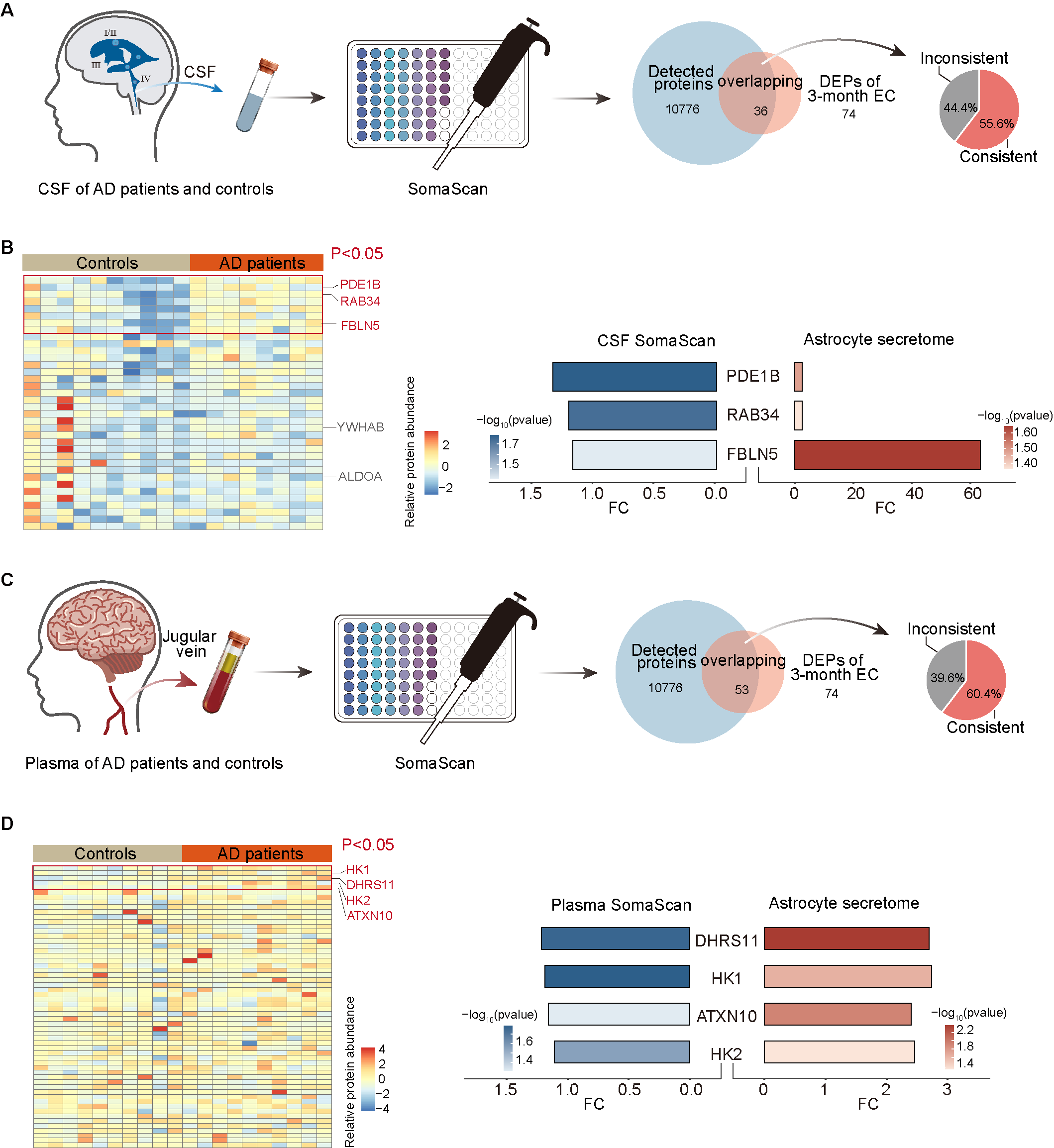

Supplement: Supplementary file 9 — Supplementary Material 9 [file 13024_2026_956_MOESM9_ESM.tif]

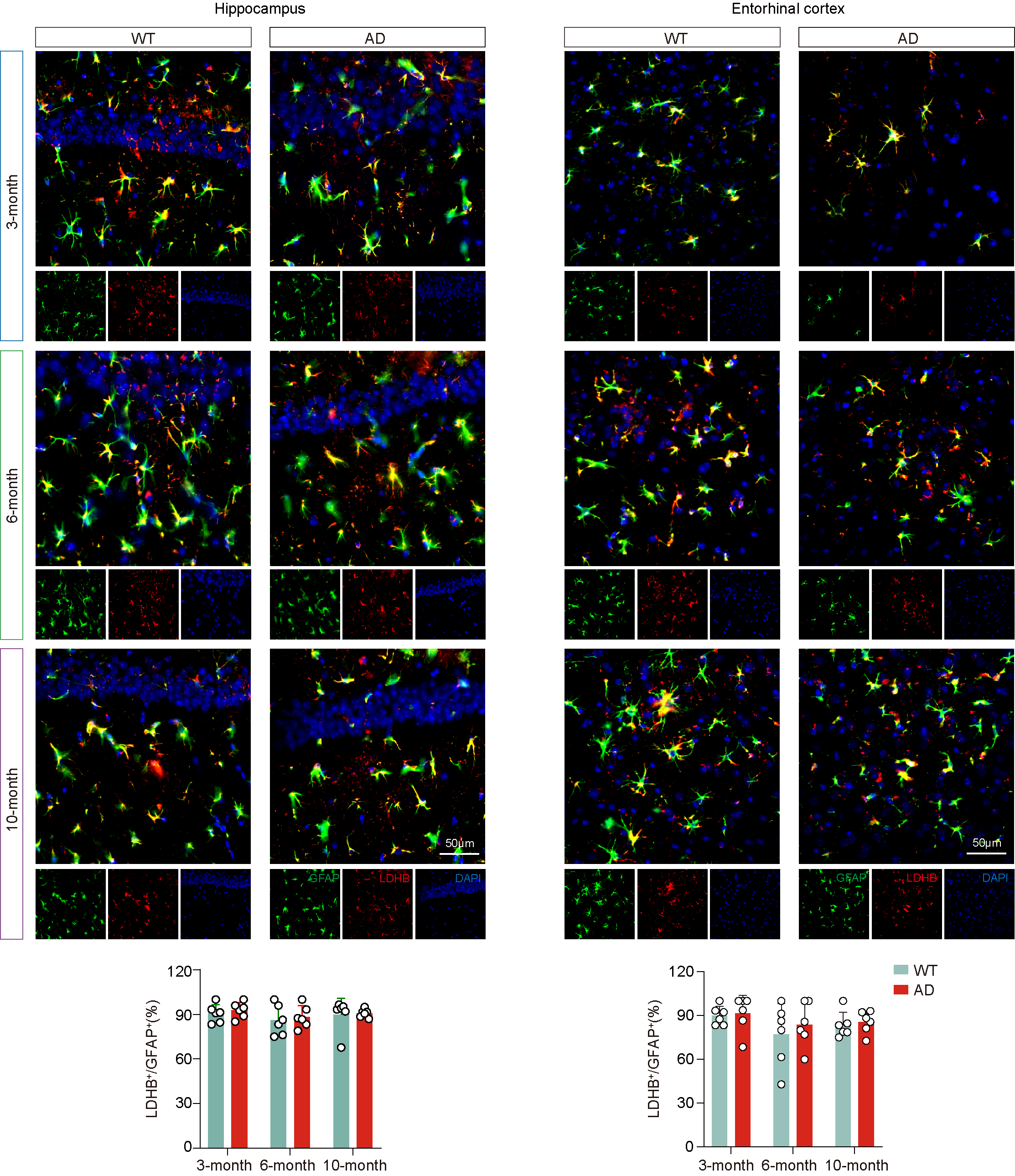

Supplement: Supplementary file 10 — Supplementary Material 10 [file 13024_2026_956_MOESM10_ESM.tif]

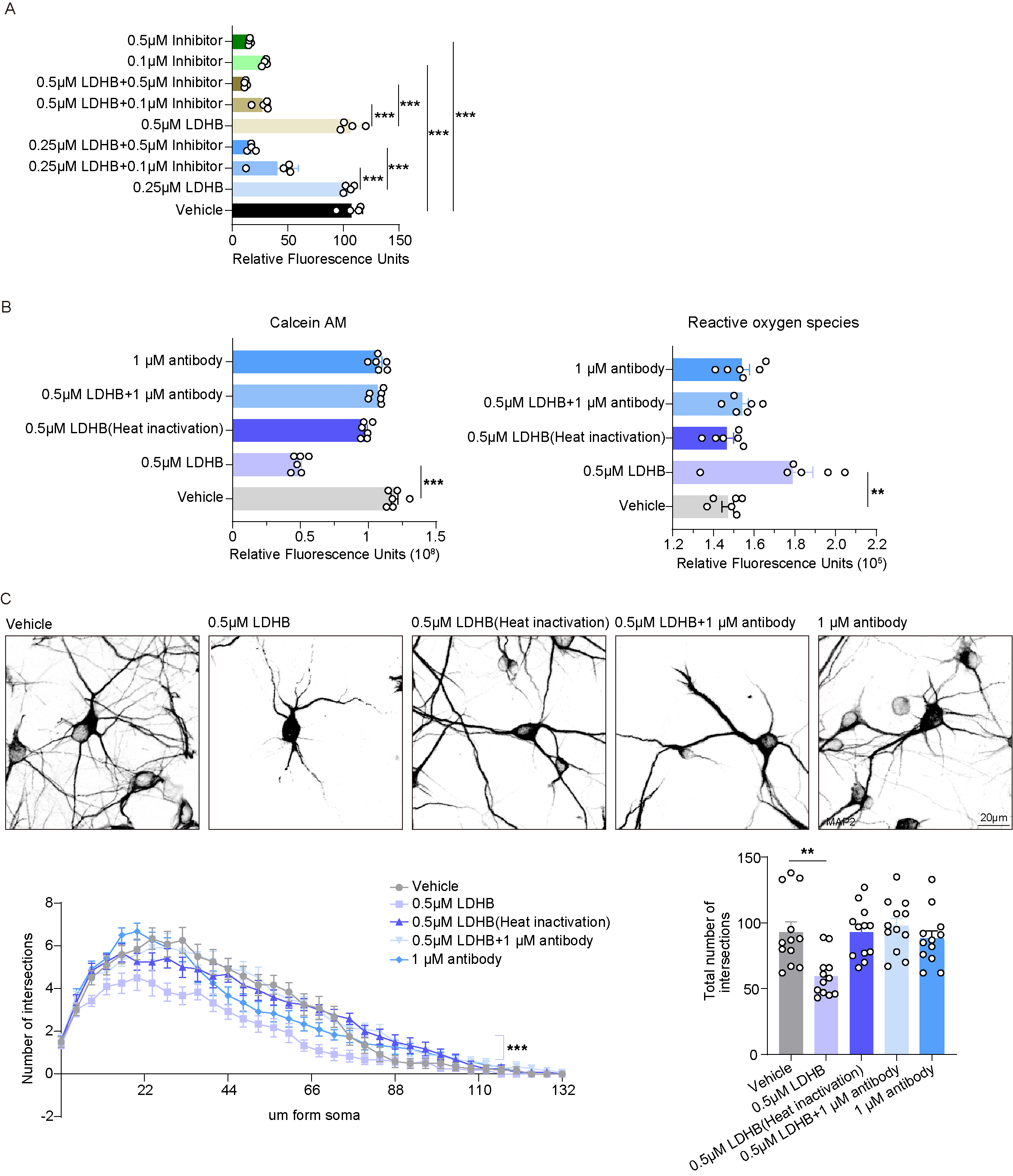

Supplement: Supplementary file 11 — Supplementary Material 11 [file 13024_2026_956_MOESM11_ESM.tif]

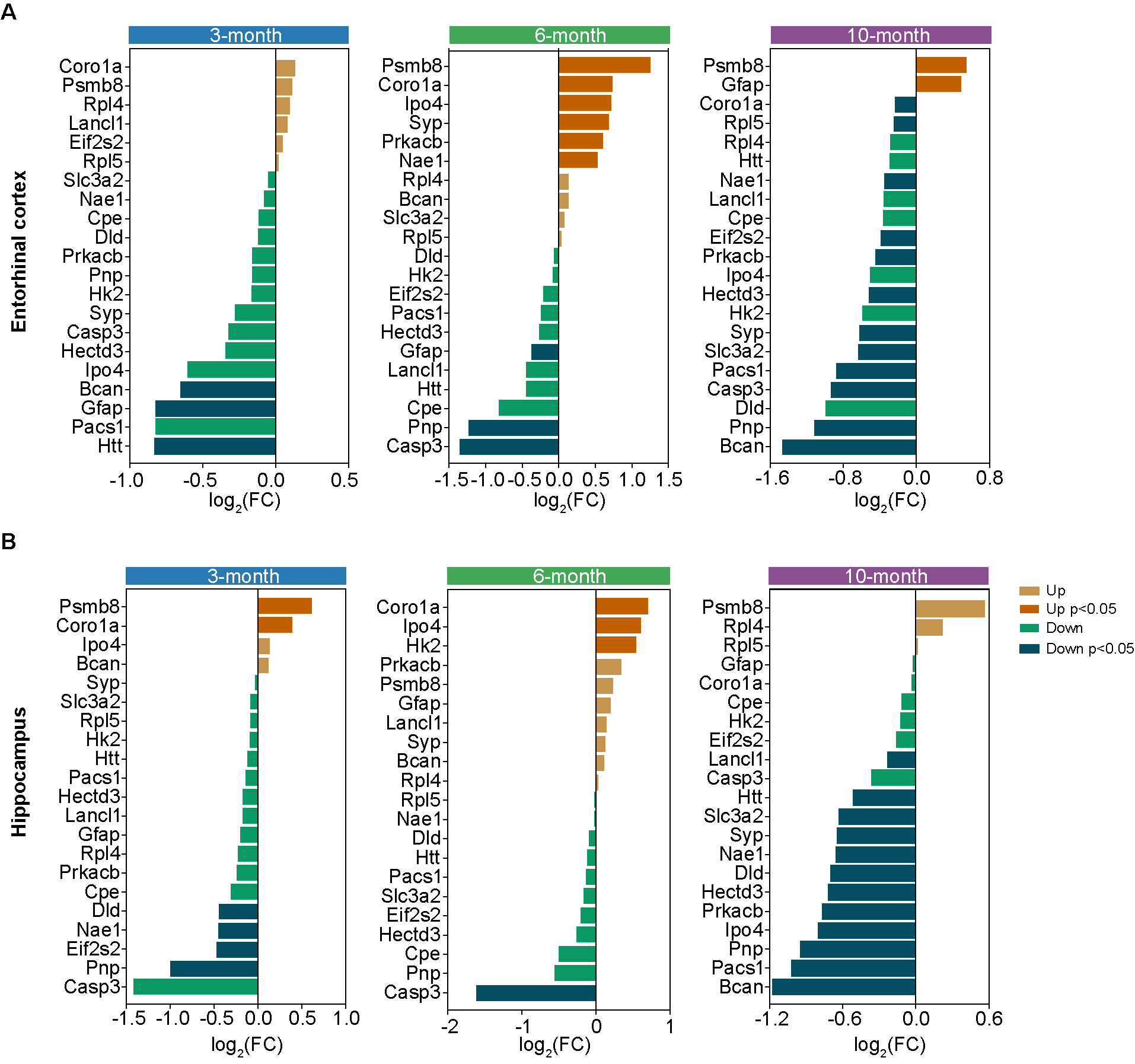

Supplement: Supplementary file 12 — Supplementary Material 12 [file 13024_2026_956_MOESM12_ESM.tif]

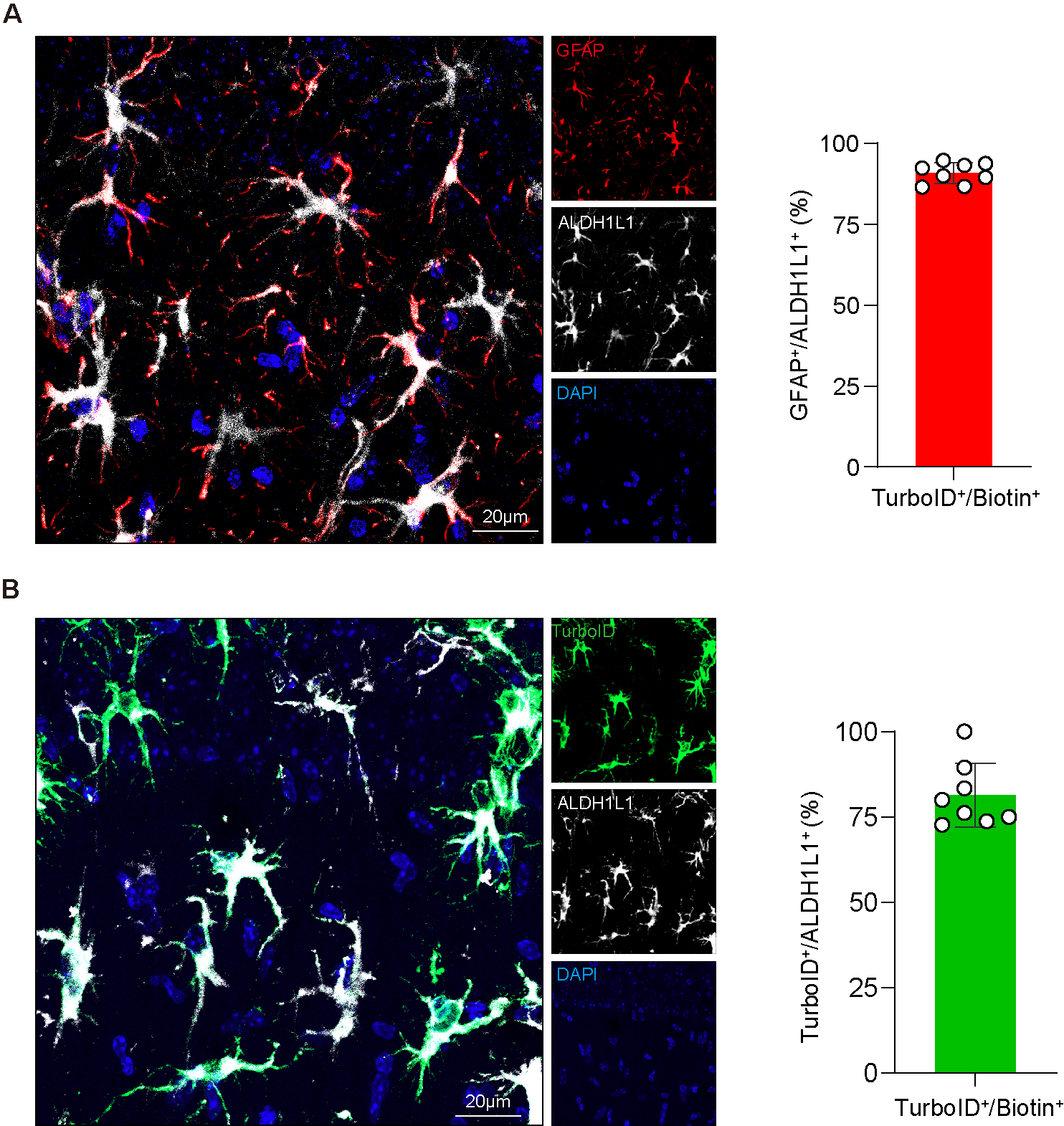

Supplement: Supplementary file 13 — Supplementary Material 13 [file 13024_2026_956_MOESM13_ESM.tif]
